# Persistent prey species in the Lotka–Volterra apparent competition system with a single shared predator

**DOI:** 10.1007/s00285-025-02184-2

**Published:** 2025-01-23

**Authors:** Hiromi Seno

**Affiliations:** Department of Computer and Mathematical Sciences, Research Center for Pure and Applied Mathematics, Graduate School of Information Sciences, Tohoku University, Aramaki-Aza-Aoba 6-3-09, Aoba-ku, Sendai, Miyagi 980-8579 Japan

**Keywords:** Apparent competition, Species invasion, Species extinction, Functional homogenization, 92D40, 92D25, 92B05, 37N25

## Abstract

We analyze the Lotka–Volterra *n* prey-1 predator system with no direct interspecific interaction between prey species, in which every prey species undergoes the effect of apparent competition via a single shared predator with all other prey species. We prove that the considered system necessarily has a globally asymptotically stable equilibrium, and we find the necessary and sufficient condition to determine which of feasible equilibria becomes asymptotically stable. Such an asymptotically stable equilibrium shows which prey species goes extinct or persists, and we investigate the composition of persistent prey species at the equilibrium apparent competition system. Making use of the results, we discuss the transition of apparent competition system with a persistent single shared predator through the extermination and invasion of prey species. Our results imply that the long-lasting apparent competition system with a persistent single shared predator would tend toward an implicit functional homogenization in coexisting prey species, or would transfer to a 1 prey-1 predator system in which the predator must be observed as a specialist (monophagy).

## Introduction

The interspecific interaction in a food web is made up of *direct* and *indirect* effects (Begon et al. 1996). Direct effect includes competition, predation and symbiosis. Indirect effect is defined as an effect on a species from another which has no direct interaction with it. The indirect effect between two species could occur through interactions with the other species in the food web. *Apparent competition* is defined by Holt (1977, 1984) as a negative indirect effect between two prey species which have a shared predator and have no direct interaction between them. Jeffries and Lawton (1984, 1985) called the corresponding indirect effect *the competition for enemy-free space*. In a system of one predator and its two prey species, one prey population plays a roll of the bioresource to increase the predator population, so that the other prey population can be regarded as *indirectly* affected by the former prey population even if no direct interaction exists between them.

There have been lots of previous ecological works related to apparent competition, in which the effect of predation on the diversity of competing prey species was mainly considered (Chaneton and Bonsall 2000; Chase et al. 2002; Frost et al. 2016; Sheehy et al. 2018; Stige et al. 2018; Gripenberg et al. 2019; Ng’weno et al. 2019). On the other hand, as Holt and Bonsall (2017) clearly describes in the up-to-date review, the “apparent competition” effect defined above has been accepted and it is used today for the theoretical studies in a variety of contexts which transcend ecology. This can be seen in the agricultural, medical and sociological sciences with a variety of examples in reality including pest control (Carvalheiro et al. 2008; Bompard et al. 2013; Jaworski et al. 2015a, b), immune dynamics (King and Bonsall 2017), and epidemics (Cobey and Lipsitch 2013) (also see the literatures cited in Holt and Lawton 1994; Holt 2023).

In nature, the members of a food web are always subjected to change on a long time scale following species extinctions and invasions (Carlton and Geller 1993; Milner-Gulland et al. 2003; Spaak et al. 2023). Morris et al. (2004) successfully demonstrated the long-term apparent competition in natural communities of herbivorous insects, and gave a suggestion that interactions mediated by shared natural enemies may be a significant factor in structuring natural communities. In lots of theoretical researches about the effect of the species extermination or introduction on the community structure, community assembly models or “global models” has been constructed, analyzed and investigated mainly to consider the stability of structure (Abrams 1996; Drossel et al. 2001; Chase et al. 2002; Fowler and Lindström 2002; Quince et al. 2005).

In contrast to almost all previous works on the population dynamics model of the shared predator(s) and two prey species with a variety of interspecific reaction under apparent competition (for example, Holt et al. 1994; Abrams et al. 1998; Schreiber 2004; McPeek 2019; Picot et al. 2019), we analyze the Lotka–Volterra *n* prey-1 predator system in which the predation is incorporated by the mass-action type of reaction term between prey and predator, while prey species have no direct interspecific interaction between them. Prey species have only indirect interactions, that is, apparent competition via the shared single predator. Focusing on the effect of apparent competition between prey species, we do not introduce any interspecific direct reaction in our modeling other than the predation between the shared single predator and every prey, differently from recent biological or theoretical/mathematical works on the apparent competition in relation to some other factors relevant to the persistence of prey species. We consider the system with a generally given per capita growth rate of prey, and derive the necessary and sufficient condition to determine which of feasible equilibria becomes asymptotically stable. Such an asymptotic stable equilibrium determines which prey species goes extinct or persists, and enables us to investigate the composition of persistent prey species at the equilibrium apparent competition system. Making use of the obtained results, we discuss the transition of such an apparent competition system with a persistent single shared predator through the extermination and invasion of prey species. Then we find that the long-term apparent competition system with a persistent single shared predator would tend to lead a kind of specific homogenization in prey species, or would transfer to a 1 prey-1 predator system in which the predator must be observed as a specialist (monophagy).

## Model

We consider the following *n* prey-1 predator system of Lotka–Volterra type with the mass-action terms for the predation:

1 $$\begin{aligned} \left\{ \begin{array}{ccl} \displaystyle \frac{dH_{i}}{dt} & =& g_i(H_{i}) H_{i} - b_{i} H_{i} P \quad (i = 1, 2, \ldots , n);\\ \displaystyle \frac{dP}{dt} & =& -\delta P + \displaystyle \sum _{i = 1}^{n} c_{i} b_{i} H_{i} P, \end{array} \right. \end{aligned}$$

where $H_i$ is the population size (e.g., density) of prey *i*, *P* the population size of predator, $b_i$ the predation rate for prey *i*, $\delta$ the predator’s natural death rate, and $c_i$ the energy conversion rate of the predation for prey *i*. The function $g_i(H_i)$ is the per capita growth rate of prey *i* when its population size is $H_i$, which is now assumed to satisfy the following features for each $i = 1, 2, \dots , n$:

- $g_i(x)$ is strictly decreasing and continuous for $x \ge 0$, and differentiable for $x > 0$;
- $$g_i(0) = r_i > 0$$;
- $g_i(K_i) = 0$ for a positive value $K_i > 0$.

The per capita growth rate of every prey follows an intraspecific negative density effect. Parameters $r_i$ and $K_i$ define the intrinsic growth rate and the carrying capacity of prey *i* respectively. One of classic choices for the function $g_i (H_i)$ is a linear one: $g_i(H_i) =r_i - \beta _i H_i$ and $K_i = r_i/\beta _i$ with the coefficient of intraspecific density effect $\beta$, then the population of prey *i* follows a sort of well-known *logistic growth* (Holt 1977; Kr̆ivan 2014; Seno et al. 2020).

In this paper, we analyze the system (1) with the general function $g_i(H_i)$ satisfying the above mathematical features. Hence note that our arguments and results in this paper are valid even when the functions $g_i (H_i)$ ($i = 1, 2,\dots , n$) are given by different formulas, as long as they all satisfy the above mathematical features. We should remark that the same system as (1) with such the general function $g_i(H_i)$ was considered primarily in Holt (1977) to a extent, and there were shown the results matching some of ours in this paper. In this sense, we are going to revisit, refine, and systematically reconsider it here with some extended concepts and hopefully wider applicability.

Prey species have no direct interspecific interaction. When the shared predator is absent, each prey population grows independently of any other prey population. Then, since $g_i(H) >0$ for any $H\in (0, K_i]$ while $g_i(H) <0$ for any $H>K_i$, it is easily seen that, when the shared predator is absent, every prey population size $H_i(t)$ from an initial value $H_i(0) > 0$ monotonically approaches $K_i$ as time passes, and $H_i(t)\rightarrow K_i$ as $t\rightarrow \infty$. Thus, as an ecologically reasonable setup, we shall consider the system (1) with the initial condition such that

2 $$\begin{aligned} P(0) > 0; \quad 0 < H_{i}(0) \le K_i \quad (i = 1, 2, \dots , n), \end{aligned}$$

since $K_i$ is the carrying capacity for prey *i*. Then, when the shared predator is absent, $H_i(t)$ monotonically increases to approach $K_i$ as time passes, and $H_i(t)\rightarrow K_i$ as $t\rightarrow \infty$. Further it is easily shown that $H_i(t) \in (0, K_i)$ and $P(t) > 0$ for any $t \ge 0$ (Appendix A):

### Lemma 1

The solution of (1) with the initial condition (2) always stays in the domain

3 $$\begin{aligned} {\mathcal {D}}:=\big \{ (H_1, H_2,\dots , H_n, P)\mid P> 0,\, 0 < H_{i}\le K_i\ (i = 1, 2, \dots , n)\big \}. \end{aligned}$$

In our model, without loss of generality, prey species are numbered in the following order, in the same way as for the model with the logistic growth of prey populations in Holt (1977); Seno et al. (2020):

4 $$\begin{aligned} \displaystyle \frac{r_1}{b_1}\ge \frac{r_2}{b_2}\ge \cdots \ge \frac{r_n}{b_n}. \end{aligned}$$

## Basic predator replacement rate

The *net replacement rate* or *net reproduction rate* is defined in ecology as the expected number of mature females produced by a mature female over its lifetime (for example, see Gotelli 2001). When it is less than one, the population size eventually decreases. This definition obviously has a correspondence to what is called *basic reproduction number* for the epidemic dynamics, which is defined as the expected number of new cases of infection caused by an infective individual in a population consisting of susceptible contacts only (for a modern review about the definition, the translation, and the practical application of basic reproduction number for the epidemic dynamics, see Delamater et al. 2019). Making use of a similar mathematical concept with the definition of basic reproduction number, we shall define here the *basic predator replacement rate* for the predator in the system (1).

Firstly for the 1 prey -1 predator system with prey species *i*

5 $$\begin{aligned} \left\{ \begin{array}{ccl} \displaystyle \frac{dH_{i}}{dt} & =& g_i(H_{i}) H_{i} - b_{i} H_{i} P;\\ \displaystyle \frac{dP}{dt} & =& -\delta P + c_{i} b_{i} H_{i} P, \end{array} \right. \end{aligned}$$

we can define the *prey-specific* basic predator replacement rate as

6 $$\begin{aligned} {\mathscr {R}}_{0, i} := \dfrac{\, 1\, }{\delta }\sup _{H_i}\big [ c_ib_iH_i\big ] = \dfrac{\, 1\, }{\delta }c_ib_iK_i, \end{aligned}$$

where $1/\delta$ gives the expected lifetime of the predator in the system (1) and (5). The prey-specific basic predator replacement rate ${\mathscr {R}}_{0, i}$ means the supremum for the number of predator’s offsprings produced by a single predator during the expected lifetime $1/\delta$ when the available prey is only of species *i*. Then, for the *n* prey-1 predator system (1), we can define the basic predator replacement rate in the same way as

7 $$\begin{aligned} {\mathscr {R}}_0^{[n]}:= \dfrac{\, 1\, }{\delta }\sup _{\{H_i\}}\displaystyle \sum _{i = 1}^{n} c_{i} b_{i} H_{i} = \sum _{i=1}^n\dfrac{\, 1\, }{\delta }c_ib_iK_i = \sum _{i=1}^{n}{\mathscr {R}}_{0, i}. \end{aligned}$$

These basic predator replacement rate is defined by the supremum for the *net* replacement rate as well as the definition of basic reproduction number in relation to the *effective reproduction number* about the epidemic dynamics (Seno 2022). The net replacement rate depends on the temporal profile of prey densities $\{ H_i\}$ in the lifespan of predator, so that it cannot be defined independently of their actual temporal variation, while it is necessarily not beyond the basic predator replacement rate.

Note that the values $\{ {\mathscr {R}}_{0, i}\}$ do not necessarily follow the order corresponding to that of prey species according to the value $r_i/b_i$ assumed by (4). For example, it is possible in our modeling that ${\mathscr {R}}_{0, 3}< {\mathscr {R}}_{0, 1}< {\mathscr {R}}_{0, 2}$ even when $r_1/b_1 \ge r_2/b_2\ge r_3/b_3$.

## Predator’s persistence

**Fig. 1:**
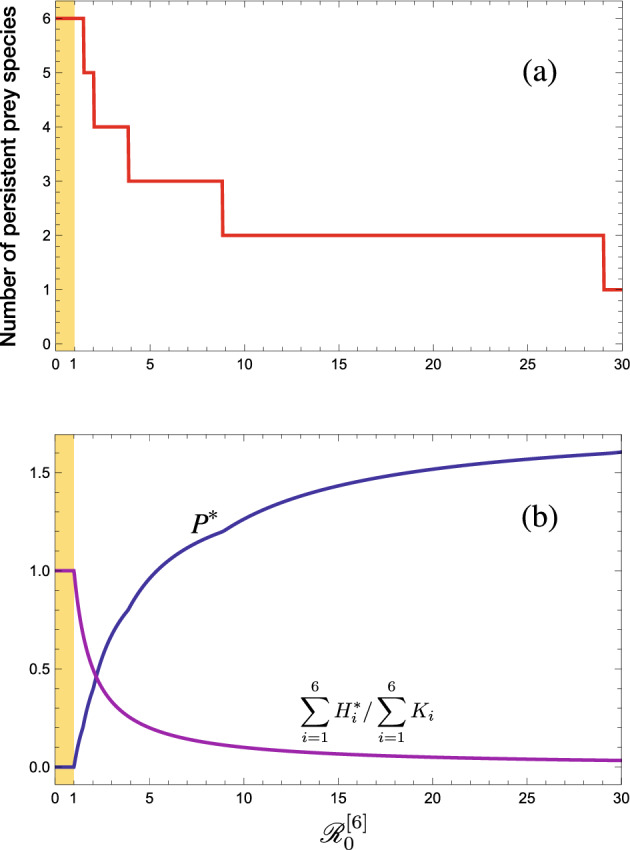
A numerical result about the $\mathscr {R}_0^{[6]}$-dependence ($\delta$-dependence) of the equilibrium after a shared predator’s invades into the system (1) with available six prey species ($n = 6$) which grow in the logistic manner: $g_i(H_i) =r_i - \beta _i H_i$ and $K_i = r_i/\beta _i$ ($i = 1, 2,\dots , 6$). (a) Number of persistent prey species at the equilibrium; (b) Equilibrium population size of predator and the relative total population size of all prey species. For ${\mathscr {R}}_0^{[6]} \le 1$, the predator goes extinct, that is, $P^* = 0$, while it coexists with all or some of prey species at the equilibrium for $\mathscr {R}_0^{[6]} > 1$. $b_{i} = 0.5$; $c_{i} = 0.1$; $\{ r_{i}\} = \{ 1.0, 0.8, 0.6, 0.4, 0.2, 0.1 \}$; $\{\beta _i\} = \{0.08, 0.06, 0.04, 0.03, 0.02, 0.01\}$; $\delta = \big ({1}/{{\mathscr {R}}_0^{[6]}}\big )\displaystyle \sum _{i=1}^nc_ib_iK_i = {3.71}/{{\mathscr {R}}_0^{[6]}}$. The largest prey-specific basic predator replacement rate is ${\mathscr {R}}_{0, 3}$

We can get the following result about the predator’s persistence for the *n* prey-1 predator system (1) (Appendix B; Fig. 1):

### Theorem 1

Predator can persist if and only if ${\mathscr {R}}_0^{[n]} > 1$. Otherwise it goes extinct.

This theorem implies that the predator tends to survive only when sufficiently many or beneficial prey species are available. On the contrary, if the available prey species are rather limited, or if all available prey species are rather poor as its foods, the predator may go extinct. Moreover the extinction of the predator is most likely to be caused by the extermination of the prey species which has the largest value of ${\mathscr {R}}_{0, i}$, since the extermination of such a prey species reduces the basic predator replacement rate ${\mathscr {R}}_0^{[n]}$ by the largest amount. Such a prey species could be regarded as a sort of “keystone species” which is the most relevant for the shared predator’s persistence. On the other hand, it is sufficient for the predator’s persistence that there is a prey species *i* with the prey-specific basic predator replacement rate ${\mathscr {R}}_{0, i}$ greater than 1, independently of how many prey species are available for the predator.

From the aspect of predator’s invadability in a habitat with *n* preys available for the predator, the condition $\mathscr {R}_{0}^{[n]} > 1$ is necessary and sufficient for the invasion success. The predator’s invasion fails if ${\mathscr {R}}_{0}^{[n]}\le 1$. In the same context, the predator’s invasion is successful if ${\mathscr {R}}_{0, i} > 1$ for a prey species *i*, while it fails only if ${\mathscr {R}}_{0, k} < 1$ for all available prey species *k*. Even when ${\mathscr {R}}_{0, i} < 1$ for all available *n* prey species in the habitat, the invasion is successful if ${\mathscr {R}}_{0}^{[n]} > 1$. This is the case where the predator invades in a habitat with a sufficient number of preys all of which however have poor quality for the predator’s reproduction.

When the predator persists, some prey species would go extinct due to the apparent competition effect as seen in the numerical example of Fig. 1 about the system (1) with six prey species growing in the logistic manner as $g_i(H_i) =r_i - \beta _i H_i$ ($i = 1, 2,\dots , 6$), considered analytically in Seno et al. (2020), and numerically in Kr̆ivan (2014). Hence it should be remarked that Theorem 1 does not necessarily show what is called *persistence* mathematically defined for the solution of system (1) (as for the mathematically defined *persistence* or related *permanence*, for example, see Hofbauer and Sigmund 1998; Thieme 2003). In the following arguments, we will focus on the feature of the system (1) with respect to which prey species goes extinct or persists with the persistent shared single predator.

## Equilibrium with persistent predator

Let us begin with considering the following type of equilibrium $E^*_{[k]}$ ($k = 1, 2,\dots , n$) for the system (1) under the condition that ${\mathscr {R}}_0^{[n]} > 1$ when the predator persists from Theorem 1:

8 $$\begin{aligned} (H_{1}, H_{2},\dots , H_{n}, P) = (H^*_{[k] , 1}, H^*_{[k] , 2},\dots , H^*_{[k] , k}, \underbrace{0,\dots , 0}_{n-k}, P^*_{[k]}) \end{aligned}$$

with $H^*_{[k], i}\in (0, K_i)$ ($i=1,2,\dots , k$) and $P^*_{[k]}>0$. From the equations of (1), the equilibrium $E^*_{[k]}$ defined as (8) can be determined by

9 $$\begin{aligned} g_i\big (H_{[k] , i}^*\big ) = b_iP^*_{[k]};\quad \displaystyle \sum _{i=1}^k\dfrac{\ H_{[k] , i}^*}{K_i}{\mathscr {R}}_{0, i}=1, \end{aligned}$$

that is,

10 $$\begin{aligned} H_{[k] , i}^* = g_i^{-1}\big (b_iP^*_{[k]}\big ) ;\quad \mathscr {G}_k(P^*_{[k]}):=\sum _{i=1}^k\dfrac{g_i^{-1}\big (b_iP^*_{[k]}\big )}{K_i}{\mathscr {R}}_{0, i}=1, \end{aligned}$$

where $g_i^{-1}$ is the inverse function of $g_i$. By the later Lemma 4 and Theorem 3 in this section, we shall show that only the equilibrium $E^*_{[k]}$ of the type given by (8) can be asymptotically stable for the system (1).

First we can prove the following lemma about the existence of the equilibrium $E^*_{[k]}$ defined by (8) (Appendix C):

### Lemma 2

The equilibrium $E^*_{[k]}$ $(k = 1, 2,\dots , n)$ defined by (8) uniquely exists in ${\mathcal {D}}$ if and only if

11 $$\begin{aligned} {\mathscr {W}}_k := {\mathscr {G}}_k\big (\frac{\, r_k}{\, b_k}\big ) = \sum _{i=1}^k\dfrac{g_i^{-1}\big (\frac{r_k/b_k}{r_i/b_i}r_i\big )}{K_i}{\mathscr {R}}_{0, i}< 1 <{\mathscr {R}}_0^{[k]} =\displaystyle \sum _{i=1}^k{\mathscr {R}}_{0, i} ={\mathscr {G}}_k(0). \end{aligned}$$

When it exists, it is satisfied that $P^*_{[k]} < r_k/b_k$.

The latter result on the equilibrium predator population size $P_{[k]}^*$ has been shown also in Holt (1977). From Lemma 2, if and only if $\mathscr {W}_n< 1 <{\mathscr {R}}_0^{[n]}$, exists the equilibrium $E^*_{[n]}$ at which all prey species persist with the shared predator. Then, making use of a Lyapunov function, we can obtain the following result on the stability of $E^*_{[n]}$ (Appendix D):

### Theorem 2

If the equilibrium $E^*_{[n]}$ exists, it is globally asymptotically stable in ${\mathcal {D}}$.

The corresponding result with prey species growing in the logistic manner as $g_i(H_i) =r_i - \beta _i H_i$ ($i = 1, 2,\dots , n$) has been shown with a Lyapunov function in Kr̆ivan (2014); Seno et al. (2020). Theorem 2 indicates that the system (1) is what is mathematically called *persistent* if the equilibrium $E_{[n]}^*$ exists as an interior equilibrium in ${\mathcal {D}}$ (as for the definition, for example, see Hofbauer and Sigmund 1998; Thieme 2003).

In contrast, we can obtain the following result on the local stability for the equilibrium $E^*_{[k]}$ with $k < n$ (Appendix E):

### Lemma 3

The equilibrium $E^*_{[k]}$ $(k < n)$ defined by (8) exists, and it is locally asymptotically stable if and only if ${\mathscr {W}}_k < 1 \le {\mathscr {W}}_{k+1}$. Moreover it is satisfied that $P^*_{[k]} \ge {r_{k+1}}/{b_{k+1}}$ at the locally asymptotically stable equilibrium $E^*_{[k]}$.

Then remark that the equilibrium $E^*_{[k]}$ $(k < n)$ exists and is unstable if ${\mathscr {W}}_{k+1} < 1$. Further we can find the following distinct result on the uniqueness of locally asymptotically stable equilibrium (Appendix F):

### Lemma 4

If ${\mathscr {W}}_k < 1 \le {\mathscr {W}}_{k+1}$ $(k < n)$, only the equilibrium $E^*_{[k]}$ can be locally asymptotically stable, while any other equilibrium in ${\mathcal {D}}$ is unstable.

Note that the results of Theorem 2, Lemmas 3 and 4 are consistent because of the non-decreasing monotonicity of the sequence $\{{\mathscr {W}}_k\}$ and the relation between ${\mathscr {W}}_k$ and ${\mathscr {R}}_0^{[k]}$ shown by Lemmas 6 and 7 in Appendix C. Therefore, these results show that a locally asymptotically stable equilibrium always and uniquely exists.

Finally we can prove the following result about the globally asymptotically stable equilibrium (Appendix G):

### Theorem 3

When ${\mathscr {R}}_0^{[n]} > 1$, globally asymptotically stable is the equilibrium $E^*_{[s]}$ in ${\mathcal {D}}$ with *s* such that

12 $$\begin{aligned} s := \max \big \{ \ell \in \{1, 2,\dots , n\}\mid {\mathscr {W}}_\ell< 1 <{\mathscr {R}}_0^{[\ell ]} \big \}. \end{aligned}$$

The result of Theorem 3 corresponds to that of Theorem 2 about the equilibrium $E^*_{[s]}$ when $s = n$ as defined by (12). However the proof of Appendix G for Theorem 3 is different from that of Appendix D for Theorem 2 in a significant point that the former needed the condition for the local stability of $E^*_{[s]}$ in order to construct a Lyapunov function.

## Which prey species goes extinct or persists

**Fig. 2:**
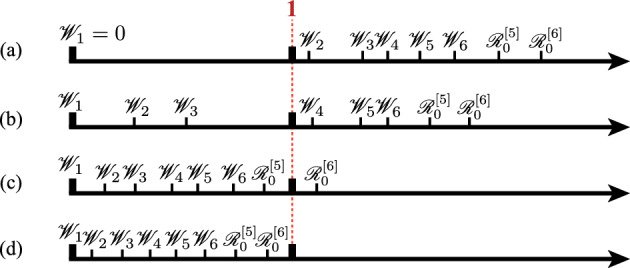
Schematic examples for the distribution of $\{{\mathscr {W}}_k\}$ that determines the persistent prey species at the equilibrium about the system (1) with available six prey species ($n = 6$). **a** Only prey species 1 persists with the persistent predator; **b** Prey species 1, 2, and 3 persist and the others go extinct with the persistent predator; **c** All prey species persist with the persistent predator; **d** Predator goes extinct and all prey species persist

From Theorems 2 and 3, we can now conclude that *the Lotka–Volterra apparent competition system* (1) *with a persistent shared single predator necessarily has a unique globally asymptotically stable equilibrium* $E^*_{[s]}$ *with* *s* *determined by* (12), *where it holds that* ${\mathscr {W}}_s< 1 \le {\mathscr {W}}_{s+1}<{\mathscr {R}}_0^{[s]}$ *if* $s < n$, and $\mathscr {W}_n< 1 <{\mathscr {R}}_0^{[n]}$ if $s = n$. This conclusion indicates that it is determined by the distribution of $\{{\mathscr {W}}_k\}$ which prey species goes extinct or persists in the Lotka–Volterra apparent competition system (1) as schematically illustrated in Fig. 2. Especially, the prey species 1 in the numbering defined by (4) must persist with the predator when the predator persists with $\mathscr {R}_0^{[n]} >1$ (Theorem 1):

### Corollary 1

When the predator persists in the system (1) with ${\mathscr {R}}_0^{[n]} >1$, prey species 1 necessarily persists with the predator.

From the definitions of *s*, ${\mathscr {W}}_s$, and $\mathscr {R}_0^{[s]}$, the number of persistent prey species *s* can be large only with relatively small values of ${\mathscr {R}}_{0, i}$ for $i = 1, 2,\dots , s$. This is because a large value of ${\mathscr {R}}_{0, \ell }$ for some $\ell$ makes the value of ${\mathscr {R}}_0^{[k ]}$ for $k \ge \ell$ large, and then the number *s* is likely to be relatively near $\ell$. Thus, roughly saying, the number of persistent prey species with a single shared predator becomes large when available prey species provide relatively small values of the basic predator replacement rate for the predator, that is, when they are relatively poor foods for the predator’s reproduction. While this result would indicate that the predator needs a number of different prey species for its persistence because those prey species are poor, it may be regarded as a consequence of the apparent competition in which the effect of apparent competition is sufficiently weak for every persistent prey species because they can keep the predator population size small with their poor contribution to the predator’s reproduction.

On the other hand, from the result of Theorem 3 with Corollary 1, we can find the following condition that all prey species except prey species 1 go extinct when the predator persists with ${\mathscr {R}}_0^{[n]} >1$ (Theorem 1):

### Corollary 2

If and only if ${\mathscr {W}}_2\ge 1$, all prey species except prey species 1 go extinct.

Remark that the condition ${\mathscr {W}}_2\ge 1$ is sufficient to have ${\mathscr {R}}_0^{[1]}> 1$ from Lemma 7 in Appendix C. The predator necessarily persists with ${\mathscr {R}}_0^{[n]}> 1$ if ${\mathscr {W}}_2\ge 1$. We note that the condition ${\mathscr {W}}_2\ge 1$ requires $r_2/b_2 < r_1/b_1$ since ${\mathscr {W}}_2 = {\mathscr {W}}_1 = 0$ if $r_2/b_2 = r_1/b_1$ as shown in this section. The condition ${\mathscr {W}}_2\ge 1$ can be equivalently written as

$$\begin{aligned} {\mathscr {R}}_{0, 1} \ge \dfrac{K_1}{g_1^{-1}\big (\frac{r_2/b_2}{r_1/b_1}r_1\big )} \quad (> 1), \end{aligned}$$

which indicates that if prey species 1 provides a sufficiently large prey-specific basic predator replacement rate for the predator, the apparent competition causes the extinction of all other prey species. It may be regarded as a consequence of the *overpredation* by the predator sustained by a particularly rich prey species (i.e., prey species 1 indexed as (4) in our modeling). At such an equilibrium, the predator appears to be a specialist (monophagy) which uses only one prey species. Such the apparent exclusion of prey species other than a particular prey species may be referred as “dynamic monophagy” (Holt and Lawton 1994; Frank van Veen et al. 2006).

As a specific case where prey species 1 follows a logistic growth with $g_1(H_1) = r_1-\beta _1H_1$ and $K_1 = r_1/\beta _1$, the above condition becomes

$$\begin{aligned} {\mathscr {R}}_{0, 1}\ge \Big (1-\dfrac{r_2/b_2}{r_1/b_1}\Big )^{-1} \quad \text{ or } \text{ equivalently } \quad \dfrac{r_2}{b_2} \le \Big (1-\dfrac{1}{{\mathscr {R}}_{0, 1}}\Big )\dfrac{r_1}{b_1}. \end{aligned}$$

**Fig. 3:**
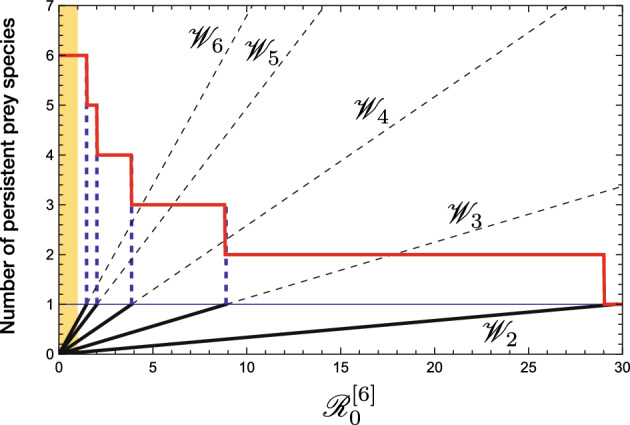
A numerical result about the $\mathscr {R}_0^{[6]}$-dependence ($\delta$-dependence) of the number of persistent prey species at the equilibrium and the values of $\{{\mathscr {W}}_k\}$, corresponding to the numerical calculation of Fig. 1 about the system (1) with available six prey species ($n = 6$)

Such a specific case is shown by the numerical example in Fig. 1, and by the corresponding numerical illustration in Fig. 3 according to the distribution of $\{\mathscr {W}_k\}$.

## Prey species of common destiny

In this section, we argue the following special feature of the system (1):

### Corollary 3

Prey species $k_1$ and $k_2$ with $r_{k_1}/b_{k_1}=r_{k_2}/b_{k_2}$ $(k_1\ne k_2)$ have a common destiny on their persistence in the system (1) with ${\mathscr {R}}_0^{[n]} > 1$ when the predator persists: They persist or alternatively go extinct together.

Now let $k_1 = \ell$, $k_2 = \ell +1$, and $r_{\ell }/b_{\ell }=r_{\ell +1}/b_{\ell +1}$. From Lemma 3, the equilibrium $E^*_{[\ell ]}$ cannot be asymptotically stable even if it exists since it does not hold that ${\mathscr {W}}_{\ell } < 1 \le \mathscr {W}_{\ell +1}$ because ${\mathscr {W}}_{\ell } = {\mathscr {W}}_{\ell +1}$ when $r_{\ell }/b_{\ell }=r_{\ell +1}/b_{\ell +1}$ (Lemma 6 in Appendix C). Hence, when the predator persists with ${\mathscr {R}}_0^{[n]} > 1$, the number of persistent prey species *s* defined by (12) must be greater or alternatively smaller than $\ell$, while *s* may be $\ell +1$. From this argument, the result of Corollary 3 holds. It is interesting that two different prey species have a common destiny on their persistence which is determined only by the value of $r_\bullet /b_\bullet$ independently of any other parameters.

For an illustrative example about this special feature, let us consider the system (1) with ${\mathscr {R}}_0^{[n]} > 1$ and

$$\begin{aligned} \displaystyle \frac{r_1}{b_1}=\frac{r_2}{b_2}=\cdots =\frac{r_\ell }{b_\ell } > \frac{r_{\ell +1}}{b_{\ell +1}}\ge \frac{r_{\ell +2}}{b_{\ell +2}}\cdots \ge \frac{r_n}{b_n}, \end{aligned}$$

where $1<\ell <n$. From Lemma 6 in Appendix C, we now have

$$\begin{aligned} {\mathscr {W}}_1 = {\mathscr {W}}_2 =\cdots ={\mathscr {W}}_\ell = 0 < \mathscr {W}_{\ell + 1}\le {\mathscr {W}}_{\ell + 2}\le \cdots \le {\mathscr {W}}_n. \end{aligned}$$

In this case, from Theorems 2 and 3 with Lemma 4, prey species 1 to $\ell$ necessarily persist, and the number of persistent prey species *s* is greater than $\ell$ if ${\mathscr {W}}_{\ell + 1} < 1$, or equal to $\ell$ if ${\mathscr {W}}_{\ell + 1} \ge 1$.

For the other example with

$$\begin{aligned} \displaystyle \frac{r_1}{b_1}> \frac{r_2}{b_2}> \cdots >\frac{r_\ell }{b_\ell } = \frac{r_{\ell +1}}{b_{\ell +1}}=\frac{r_{\ell +2}}{b_{\ell +2}}\cdots =\frac{r_n}{b_n}, \end{aligned}$$

we have

$$\begin{aligned} {\mathscr {W}}_1< {\mathscr {W}}_2<\cdots <{\mathscr {W}}_\ell = \mathscr {W}_{\ell + 1}= {\mathscr {W}}_{\ell + 2}=\cdots ={\mathscr {W}}_n. \end{aligned}$$

In this case, prey species 1 necessarily persist from Corollary 2, and the number of persistent prey species *s* is less than $\ell$ if ${\mathscr {W}}_{\ell } \ge 1$, or equal to *n* if ${\mathscr {W}}_{\ell } < 1$ because of Theorem 2 with Lemma 2.

As the extremal case, we may consider the system (1) with ${\mathscr {R}}_0^{[n]} > 1$ and $r_i/b_i = r/b$ for all $i = 1, 2,\dots , n$. Since ${\mathscr {W}}_k = 0$ for all $k = 1, 2,\dots , n$, Theorem 2 with Lemma 2 indicates that all available prey species persist with the persistent predator, which can be regarded as the case of $s = n$.

## State transition by prey extermination/invasion

In this section, we consider the state transition by the extermination of a persistent prey species or by the invasion of an alien prey species from an asymptotically stable coexistent equilibrium with the persistent predator and more than one persistent prey species. In this paper, the ‘extinction’ means a consequence of the population dynamics between prey and predator, whereas the ‘extermination’ may be caused by the other kinetics, for example, by a human activity (e.g., harvesting, culling, or pollution) or by a stochastic ecological disturbance/disaster (e.g., tempest, epidemics, or fire). In a more mathematical sense, the Mextermination’ of a prey species results in the reduction of the dimension of system from $n+1$ to *n*, while the ‘extinction’ must be necessarily considered for the system (1) of $n+1$ dimension. In contrast, the ‘invasion’ of an alien prey species results in the increase of the dimension of system from *n* to $n+1$, and then the population dynamics follow the system (1) of $n+1$ dimension.

**Fig. 4:**
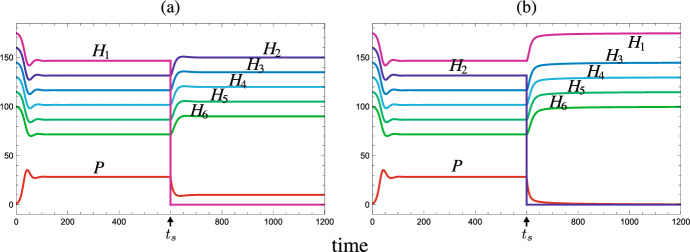
Temporal variation of population sizes after the extermination of a prey species at $t=t_s=600$ from the coexistent equilibrium with a shared predator and six prey species which grow in the logistic manner: $g_i(H_i) =r_i - \beta _i H_i$ and $K_i = r_i/\beta _i$ ($i = 1, 2,\dots , 6$). (a) Prey $H_1$ is exterminated. No secondary extinction occurs; (b) Prey $H_2$ is exterminated. The shared predator goes extinct after the extermination. $\delta = 0.48$; $b_{i} = 0.001$; $\beta _{i} = 0.001$; $\{c_{i}\} = \{{0.5}, 0.8, 0.8, 0.8, 0.8, 0.8\}$; $\{ r_{i}\} = \{ 0.175, 0.16, 0.145, 0.13, 0.115, 0.1 \}$; $\{{\mathscr {R}}_{0,i}\} = \{0.1823,{0.2667},0.2417,0.2167,0.1917,0.1667 \}$; $\{{\mathscr {W}}_i\} = \{0,0.0156,0.0563,0.1219,0.2125,0.3281\}$; ${\mathscr {R}}_0^{[6]} = 1.2656$; $H_{i}(0)=r_i/\beta _i$; $P(0) = 1.0$

First we can obtain the following theorem on the influence of a prey species extermination (Appendix H):

### Theorem 4

If a prey species is exterminated from an asymptotically stable coexistent equilibrium, the system transfers to a state at which the predator coexists with the rest of prey species or alternatively goes extinct.

This theorem indicates that the extermination of a prey species from the coexistent equilibrium does not cause any secondary extinction of other prey species even with the apparent competition. As numerically exemplified by Fig. 4, it depends on the prey-specific basic predator replacement rate of which prey species’ extermination can lead to the extinction of predator. The prey species with a large prey-specific basic predator replacement rate could be the keystone prey species for the predator’s persistence, as indicated by Theorem 1. It does not necessarily match the order of prey species defined by (4).

**Fig. 5:**
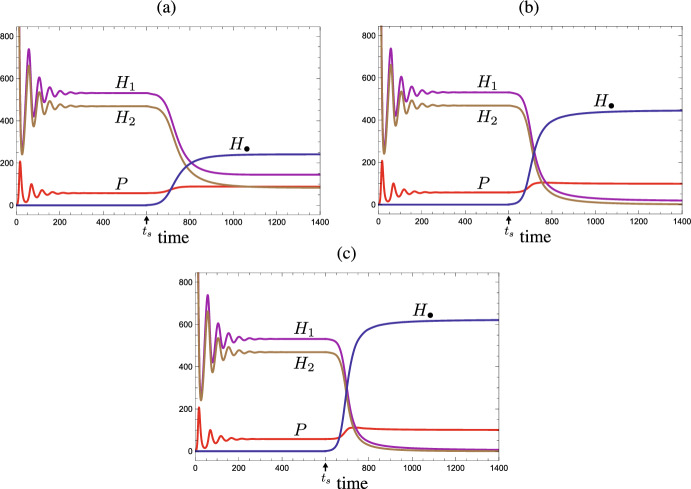
Temporal variation of population sizes after the invasion of an alien prey species $H_\bullet$ at $t=t_s=600$ into the coexistent equilibrium with the shared predator and two native prey species. Every prey population grows in the logistic manner: $g_i(H_i) =r_i - \beta _i H_i$ and $K_i = r_i/\beta _i$ ($i = 1, 2, \bullet$). Numerical calculations with $b_1 = b_2 = 0.001$; $\delta = 0.3$; $\beta _1=\beta _2=\beta _\bullet = 0.00008$; $c_1 = c_2 = 0.3$; $c_\bullet = 1.2$; $r_{1} = 0.1$; $r_{2} = 0.095$; $r_\bullet = 0.09$; ${\mathscr {R}}_{0,1} = 1.25$; ${\mathscr {R}}_{0,2} = 1.1875$; ${\mathscr {R}}_0^{[2]} = 2.4375$; ${\mathscr {W}}_1 = 0$; ${\mathscr {W}}_2 = 0.0625$; $H_{1}(0) = 1250.0$; $H_{2}(0) = 1187.5$; $H_\bullet (t_s) = 1.0$; $P(0) = 1.0$. (a) No extinction occurs by the alien prey species of $b_{\bullet } = 0.0008$ (${\mathscr {R}}_{0,\bullet } = 3.6$); (b) Only native prey $H_2$ goes extinct by the alien prey species of $b_{\bullet } = 0.00055$ (${\mathscr {R}}_{0,\bullet } = 2.475$); (c) All native prey populations $H_1$ and $H_2$ go extinct by the alien prey species of $b_{\bullet } = 0.0004$ (${\mathscr {R}}_{0,\bullet } = 1.8$)

Next, we consider the state transition by the invasion of an alien prey species in the coexistent equilibrium with a shared predator and its native prey species. From Theorems 2 and 3, we find that the system may transfer to one of the following four states after the invasion of an alien prey species (see Fig. 5):

- The alien prey goes extinct, and the system returns to the original state.
- No native prey species goes extinct, and the predator coexists with them and the alien prey species.
- Some native prey species go extinct, and the predator coexists with the other surviving native and the alien prey species.
- All native prey species go extinct, and the predator coexists only with the alien prey species.

Note that the invasion failure with the extinction of alien prey is induced by the predation pressure from the native predator population sustained by the native prey populations, if the alien prey species could not have an interspecific relation like competitive to the native prey species at least at the stage of its invasion.

We can prove the following theorem for the influence of the invasion of an alien prey species on the apparent competition system (1) with *n* native prey species (Appendix I):

### Theorem 5

If invades an alien prey species with parameters $r_\bullet$, $K_\bullet$, $b_\bullet$, $c_\bullet$, ${\mathscr {R}}_{0,\bullet } = c_\bullet b_\bullet K_\bullet /\delta$, and function $g_\bullet$ in the asymptotically stable coexistent equilibrium $E_{[n]}^*$ of the system (1), the state transfers to the following equilibrium from the asymptotically stable equilibrium $E_{[n]}^*$:

$$\triangleright $$
The alien prey species goes extinct, and the system returns to $E^*_{[n]}$ if and only if
  13 $$\begin{aligned} \dfrac{r_\bullet }{b_\bullet }< \dfrac{r_n}{b_n} \ \text{ and } \ {\mathscr {G}}_n\big (\frac{\, r_\bullet }{\, b_\bullet }\big ) = \displaystyle \sum _{i=1}^n\dfrac{g_i^{-1}\big (\frac{r_\bullet /b_\bullet }{r_i/b_i}r_i\big )}{K_i}{\mathscr {R}}_{0, i} \ge 1. \end{aligned}$$
$$\triangleright $$
Every native prey species $\ell$ satisfying the following condition becomes extinct
  14 $$\begin{aligned} \dfrac{r_\ell }{b_\ell }<\dfrac{r_\bullet }{b_\bullet } \quad \text{ and }\quad 1-\dfrac{g_\bullet ^{-1}\big (\frac{r_{\ell }/b_{\ell }}{r_\bullet /b_\bullet }r_\bullet \big )}{K_\bullet }{\mathscr {R}}_{0, \bullet } \le {\mathscr {W}}_{\ell }, \end{aligned}$$
  while the alien prey species persists. Especially if native prey species $\ell = 1$ satisfies the condition (14), all native prey species become extinct while the alien prey species persists.
$$\triangleright $$
The alien prey species and all native prey species coexist if and only if
  15 $$\begin{aligned} \dfrac{r_\bullet }{b_\bullet }< \dfrac{r_n}{b_n} \ \text{ and } \ {\mathscr {G}}_n\big (\frac{\, r_\bullet }{\, b_\bullet }\big ) < 1, \end{aligned}$$
  or alternatively
  16 $$\begin{aligned} \dfrac{r_\bullet }{b_\bullet }\ge \dfrac{r_n}{b_n} \ \text{ and } \ 1-\dfrac{g_\bullet ^{-1}\big (\frac{r_{n}/b_{n}}{r_\bullet /b_\bullet }r_\bullet \big )}{K_\bullet }{\mathscr {R}}_{0, \bullet } >{\mathscr {W}}_{n}. \end{aligned}$$

Since this theorem assumes the invasion of an alien prey species in the asymptotically stable coexistent equilibrium $E_{[n]}^*$ of the system (1), it should be considered under the condition that ${\mathscr {W}}_n< 1 <{\mathscr {R}}_0^{[n]}$ from Lemma 2 and Theorem 2.

As a result from Theorem 5, the invasion of an alien prey species with ${r_\bullet }/{b_\bullet }\le {r_n}/{b_n}$ does not cause the extinction of any native prey species. Only the invasion of an alien prey species with ${r_\bullet }/{b_\bullet }>{r_n}/{b_n}$ may cause the extinction of some native prey species. Hence the apparent competition system with large values of $r_i/b_i$ for all native prey species could be highly resistant to the invasion of alien prey species. Only an alien prey species with a sufficiently large value of $r_\bullet /b_\bullet$ can succeed in the invasion to such an apparent competition system, so that such a successful invasion of alien prey species would be rare in an ecological sense. For this reason such a system could be regarded as being at a quasi-climax state as the apparent competition system.

**Fig. 6:**
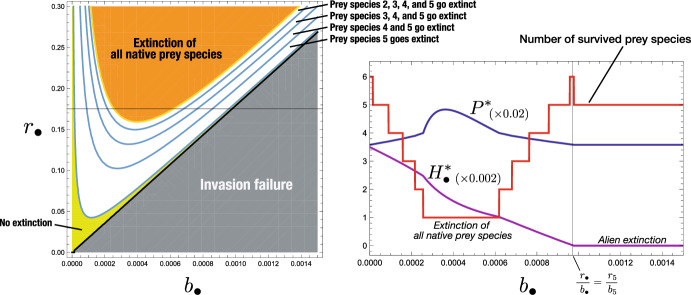
Numerical calculation on the $(b_\bullet , r_\bullet )$-dependence of the equilibrium after the invasion of an alien prey species into the system at the coexistent equilibrium with a predator and five native prey species. All prey populations grow in the logistic manner: $g_i(H_i) =r_i - \beta _i H_i$ and $K_i = r_i/\beta _i$ ($i = 1, 2,\dots , 5, \bullet$). The left figure shows the dependence of the persistence of native prey species on the parameters. The right figure shows the $b_\bullet$-dependence of the number of surviving prey species, the equilibrium population sizes of the alien prey species $H_\bullet$ and the predator $P^*$ for $r_\bullet = 0.175$. Commonly, $\beta _\bullet = 0.0001$; $c_\bullet = 1.2$; $\delta = 0.38$; $b_{i} = 0.001$; $\beta _{i} = 0.0001$; $c_{i} = 0.7$; $\{ r_{i}\}=\{ 0.2, 0.195, 0.19, 0.185, 0.180 \}$; $\{{\mathscr {R}}_{0,i}\} = \{3.6842,3.5921,3.5,3.4079,3.3158\}$; $\{{\mathscr {W}}_i\} = \{0,0.0921,0.2763,0.5526,0.9210\}$; $\mathscr {R}_0^{[5]} = 17.5$

Figure 6 shows a numerical example about the state transition by the invasion of an alien prey species. As indicated by Theorem 5, the structure of the system at the equilibrium state newly established by the successful invasion of an alien prey species could sensitively depend on the characteristics of the alien prey species. Especially we see that the alien prey species with a larger intrinsic growth rate $r_\bullet$ is more likely to cause the extinction of a greater number of native prey species. The coexistence of alien prey species with all native prey species is little likely unless the successfully invading alien prey species has a sufficiently small value of ${r_\bullet }/{b_\bullet }$ or that sufficiently similar to the smallest one of native prey species (i.e., $r_n/b_n$).

## Equilibrium predator population size

From Lemmas 2 and 3, we have the following result on the predator population size at the asymptotically stable equilibrium when it persists:

### Corollary 4

When the predator persists in the system (1) with ${\mathscr {R}}_0^{[n]} > 1$, the predator population size $P_{[s]}^*$ at the asymptotically stable equilibrium $E^*_{[s]}$ satisfies that $P_{[s]}^*\in [\, {r_{s+1}}/{b_{s+1}}, {r_{s}}/{b_{s}}\, )$ if $s<n$, and $P_{[n]}^*<{r_{n}}/{b_{n}}$ if $s = n$ with the number *s* defined as (12).

This result briefly shows that the predator population size at the asymptotically stable equilibrium is upper-bounded by the smallest value of $r_i/b_i$ for coexisting prey species, that is, $r_s/b_s$.

To understand more how the predator population size changes after the state transition caused by the extermination or invasion of a prey species, we compare the equilibrium predator population sizes before and after the state transition. First we can obtain the following result about the predator population size at the equilibrium transferred from the coexistent equilibrium by the extermination of a prey species (Appendix J):

### Theorem 6

By the extermination of a prey species *k* from the coexistent equilibrium $E^*_{[n]}$ for the system (1), the system transfers to an equilibrium at which the predator population necessarily has a size smaller than before. Simultaneously every surviving prey population at the newly established equilibrium has a size greater than before.

The last part of this theorem can be easily seen from (10) because of the decreasing monotonicity of $g^{-1}$ when the equilibrium predator population size becomes smaller. A numerical example is seen in Fig. 4.

Next, from Theorem 6, we can obtain the following lemma about the equilibrium predator population size after the successful invasion of an alien prey species without the extinction of any native prey species:

### Lemma 5

When an alien prey species successfully invades in the system (1) at the asymptotically stable equilibrium $E^*_{[n]}$ and does not cause the extinction of any native prey species, the predator population size gets larger at the newly established equilibrium $E^*_{[n\oplus 1]}$ than before the invasion.

This is because such a successful invasion of an alien prey species without the extinction of any native prey species corresponds to the state transition from the asymptotically stable equilibrium $E_{[n]}^*$ to the asymptotically stable equilibrium $E_{[n\oplus 1]}^*$ with all native and an alien prey species. Then it is the reverse transition from $E_{[n\oplus 1]}^*$ to $E_{[n]}^*$ by the extermination of the alien prey species at the coexistent equilibrium $E_{[n\oplus 1]}^*$, which has been considered in Theorem 6, where it is shown that $P_{[n\oplus 1]}^*>P_{[n]}^*$.

As a consequence, making use of Corollary 4 and the other nature of the system (1), we can prove the following theorem on the change of equilibrium predator population size caused by the successful invasion of an alien prey species in the system (1) (Appendix K):

### Theorem 7

The successful invasion of an alien prey species always results in an increase of the predator population size, independently of how many native prey species are extinct at the new equilibrium. As the number of extinct native prey species gets larger, the predator population size becomes greater at the new equilibrium than before the invasion.

This feature of the system (1) is numerically illustrated in Figs. 1, 5, and 6. As the predator population size increases, each of surviving native prey populations naturally has a size smaller at the new equilibrium than before. This can be easily seen from (10).

As indicated by Fig. 6, we can further prove the following feature of the system (1) with respect to the predator population size $P^*_{[\bullet ]}$ the equilibrium $E^*_{[\bullet ]}$ where the predator coexists with only the alien prey species after the extinction of all native prey species (Appendix L):

### Corollary 5

At the asymptotically stable equilibrium $E^*_{[\bullet ]}$ where the predator coexists with only an alien prey species of parameters $r_\bullet$, $K_\bullet$, $b_\bullet$, $c_\bullet$, $\mathscr {R}_{0,\bullet }$, and function $g_\bullet$, the equilibrium predator population size $P^*_{[\bullet ]}$ takes the maximum for a specific value of $b_\bullet$.

For the specific model with one native and an alien prey species which follow the logistic growth of population size, $g_j(H_j) =r_j - \beta _j H_j$ and $K_j = r_j/\beta _j$ ($j = 1, \bullet$), we have

$$\begin{aligned} P^*_{[\bullet ]} = \dfrac{1}{\ b_\bullet }\bigg (r_\bullet -\frac{\beta _\bullet \delta }{\ c_\bullet }\,\frac{1}{\ b_\bullet }\bigg ), \end{aligned}$$

and then the condition (14) with $\ell = 1$ becomes

17 $$\begin{aligned} \dfrac{\ r_\bullet ^2}{4}\dfrac{c_\bullet }{\beta _\bullet \delta }\ge \dfrac{\, r_1}{\, b_1} \quad \text{ and }\quad b_\bullet \in (b_\bullet ^-, b_\bullet ^+) \end{aligned}$$

with

$$\begin{aligned} b_\bullet ^{\pm } := \dfrac{r_\bullet \pm \sqrt{r_\bullet ^2-4(\beta _\bullet \delta /c_\bullet )(r_1/b_1)\, }}{2r_1/b_1}. \end{aligned}$$

At the asymptotically stable equilibrium $E^*_{[\bullet ]}$ which satisfies the condition (17), it can be easily shown that $P^*_{[\bullet ]}$ takes the maximum $\beta _\bullet \delta /c_\bullet$ for $b_\bullet = 2\beta _\bullet \delta /(r_\bullet c_\bullet )\in (b_\bullet ^-, b_\bullet ^+)$, which corresponds to ${\mathscr {R}}_{0,\bullet } = 2$.

## Concluding remarks

In this paper, we analyzed the Lotka–Volterra *n* prey-1 predator apparent competition system (1), focusing on which prey species goes extinct or persists. We have shown that the extinct prey species has the smaller value of *r*/*b* than that of persistent prey species in our model (Theorem 3).

**Basic predator replacement rate of available prey species**

The predator goes extinct if every available prey species provides very small basic predator replacement rate for the predator (${\mathscr {R}}_{0, i}$ defined by (6)) (Theorem 1). When a prey species provides a sufficiently large basic predator replacement rate, the predator persists and some of available prey species may go extinct due to the effect of apparent competition. In such a case, if all other prey species provide very small basic predator replacement rates, the predator’s persistence relies on the prey species with a sufficiently large basic predator replacement rate. Then such the prey species can be regarded as the “keystone species” for the predator’s persistence, because the extinction of the prey species by an ecological disturbance for example could cause the predator’s extinction to make the collapse of the apparent competition system (see Fig. 4b). Such an apparent competition system could be regarded as little sustainable. In the context of a pest control, in order to suppress/eliminate the pest population regarded as a generalist predator, it would be a good option to identify such a keystone prey species for the pest. In contrast, if there are some available prey species with large basic predator replacement rate, the apparent competition system would be less vulnerable to the extinction of available prey species with some cause.

**Invasion of alien prey species**

Successful invasion of an alien prey species could strengthen the apparent competition effect on native prey species. Then, some native species would go extinct, and the system would transfer to the equilibrium with the predator, the alien prey species and persistent native prey species (Theorem 5). Thus, a series of the invasion of alien prey species could cause the decrease in the number of prey species available for the predator due to the extinction of prey species by the apparent competition. We note that the persistent prey species must have sufficiently large value of *r*/*b*, while the extinct prey species have the value smaller than that of the alien prey species.

Independently of how many native prey species go extinct with the successful invasion of an alien prey species, the equilibrium predator population size becomes larger than before after the established settlement of the new prey species (Theorem 7). Then the equilibrium population size of every native prey species becomes smaller than before, since the increased size of predator population makes the predation pressure stronger for them. Indeed Messelink et al. (2010) investigated the biological control by a generalist predator in a laboratory system of three pest species (Western flower thrips, greenhouse whitefly, and spider mite) and their predator (predatory mite), in which such a dependence of the predator population size on the composition of prey species was clearly observed.

The predator population size becomes larger as the number of persistent native prey species gets smaller after a successful invasion of an alien prey species. As the number of extinct prey species due to the successful invasion of an alien prey species gets larger, the persistent native prey species undergo stronger apparent competition effect. In such a case, the apparent competition effect from the alien prey species would overcompensate that from those native prey species which have gone extinct. In short, the successful invasion of an alien prey species which could cause a strong apparent competition effect is likely to result in the extinction of native prey species. From the viewpoint of the predator, such extinction of native prey species appears as an exchange of some available prey species with the other species preferable for the predator’s reproduction.

As a consequence, the invasion of an alien prey species into the equilibrium of an apparent competition system never reduces the predator population size, and its success necessarily makes the effect of apparent competition stronger to the native prey species. In contrast, the extinction of a prey species from the equilibrium of an apparent competition system never causes any secondary extinction of the other prey species, while it may cause the extinction of predator (Theorem 4). These results may be regarded as corresponding to those given by Petchey (2000), who investigated some microcosms of bacteria and bacterivores in laboratory and showed that the prey diversity can affect the predator population dynamics. As implied by our theoretical arguments above, the apparent competition could be a significant factor to determine the structure of foodweb, tangled with the other interspecific reactions within it, as discussed in Holt and Lawton (1994); Chaneton and Bonsall (2000); Frost et al. (2016); Sheehy et al. (2018); Stige et al. (2018); Gripenberg et al. (2019); Ng’weno et al. (2019).

In the context of pest control, the invasion of alien prey species would be effective only if the purpose of the pest control is to reduce the population of a native prey species (which is the pest), while the extermination of a native prey species would be effective only if the purpose is to reduce the predator population (which is the pest) as already mentioned in the above. Actually for biological control in agroecosystems, the invasion of an alien species would be a better choice compared to the extermination of a native species. In grape vineyards, Karban et al. (1994) found that the release of economically unimportant Willamette mites alone, or of predatory mites alone fails to significantly reduce populations of the damaging Pacific spider mite. However, when both herbivorous Willamette and predatory mites were released together, the population of the Pacific mites was reduced. This may be regarded as a case when the invasion of an alien prey species (the herbivorous Willamette mite) would be effective to reduce a native prey population (the Pacific mite) if there is a shared predator (the predatory mite). Another similar experimental research was conducted by Liu et al. (2006), and concluded the effectiveness of application of shared predator and apparent competitor for the pest control. In contrast to the pest control, the apparent competition could be an important dynamical factor in the context of the conservation of endangered prey species too (DeCesare et al. 2010). As Holt and Hochberg (2001) discussed, the indirect interactions may contribute to the biological control in such a way. At the same time, it should be kept in mind that the invasion of an alien species as a biological control agent would cause the decrease of some native species populations other than that of the target pest species (Carvalheiro et al. 2008).

**Functional homogenization**

**Fig. 7:**
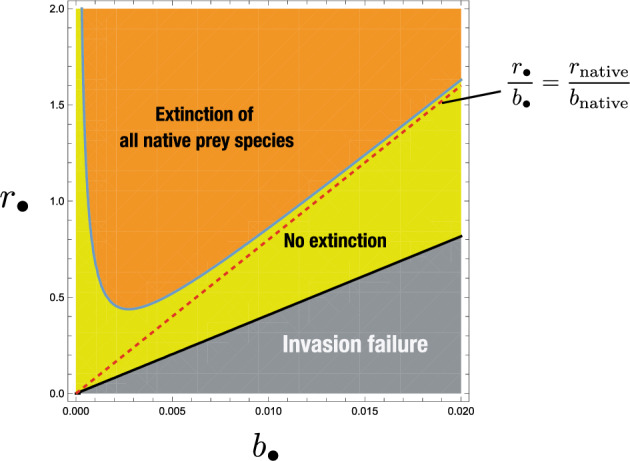
Numerical calculation on the $(b_\bullet , r_\bullet )$-dependence of the equilibrium after the invasion of an alien prey species into the system at the coexistent equilibrium with a predator and six native prey species with the same value $r_k/b_k = 80.0$ ($k = 1, 2,\dots , 6$). All prey populations grow in the logistic manner: $g_i(H_i) =r_i - \beta _i H_i$ and $K_i = r_i/\beta _i$ ($i = 1, 2,\dots , 5, \bullet$). $\beta _{k} = 0.001$; $\{ r_{k}\} = \{0.32,0.16,0.08,0.04,0.02,0.01\}$; $\{ b_k\} = \{0.004,0.002,0.001,0.0005,0.00025,0.000125\}$; $\{c_{k}\} = \{ 0.5, 0.8, 0.8, 0.8, 0.8, 0.8\}$; $\delta = 0.48$; $\{{\mathscr {R}}_{0,k}\} = \{1.3333,0.5333,0.1333,0.0333,0.0083,0.0021\}$; ${\mathscr {R}}_0^{[6]} = 2.0438$; ${\mathscr {W}}_k = 0$; $\beta _\bullet = 0.001$; $c_\bullet = 0.8$

As a special case in our model, if every prey species has a common value of *r*/*b*, the number of prey species with which the shared predator can coexist is unlimited for the Lotka–Volterra *n* prey-1 predator system (1), * independently of the difference not only in the values of* *r* *and* *b* *themselves but also in any other parameter * (Corollary 3). However, such an apparent competition system would be highly vulnerable to the invasion of an alien prey species. As indicated by the illustrative numerics in Fig. 7 about the system transition by the invasion of an alien prey species, the invasion success results in the coexistence with all native species or alternatively the extinction of all native prey species. In the latter case, the system transfers to that of 1 prey-1 predator where the prey is the successfully settled alien.

**Fig. 8:**
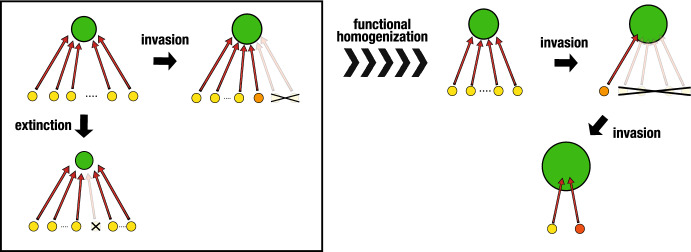
A scenario for the long-term transition of apparent competition system toward the functional homogenization with a smaller number of prey species, driven by the extinction of native prey species and the invasion of alien prey species. For detail, see the main text

These results imply that the long-lasting existence of an apparent competition system undergoing a number of alien prey invasions may lead to the relatively large value of *r*/*b* for its persistent prey species. Further, since the value *r*/*b* must have an upper bound for some biological restriction, the variance of *r*/*b* over the persistent prey species would necessarily become small as long as the system remains an apparent competition system even after a sequence of changes in the member of prey species following their extinction and invasion (see Fig. 8). This was discussed also in Holt (1977, 1984); Holt and Bonsall (2017) as the high species diversity under the condition that the value of *r*/*b* is similar for all prey species.

Furthermore this result may be related to the “biotic homogenization”, the process making the species composition more similar after the alien species invasion, as Dangremond et al. (2010) discussed about the plant community with relation to the apparent competition. Since our results indicate that only the values *r*/*b* representing the nature of persistent prey species come to have a small variance in the apparent competition system of our model, it should be specifically regarded as *functional homogenization* defined in Olden (2006); Olden and Rooney (2006). Although it might be accompanied with “genetic homogenization”, our results does not imply it for the transition of apparent competition system.

As a similar theoretical work by numerics with a specific mathematical model, Spaak et al. (2023) considered the assembly of two-trophic-level ecosystem with a series of invasions and extinctions. They discussed the trait distribution of prey and predator species, and got the results that the trait distribution of prey species mimicked a given resource distribution for them, while that of predator species tended to follow the trait distribution of prey species. Their results may be also regarded as on such a functional homogenization by the system transition with a series of invasions and extinctions.

On the other hand, Clavel et al. (2011) discussed a global functional homogenization with the world wide decline of specialist species. It is caused by the ecological disturbance with the habitat destruction, degradation in a global scale. They argues the higher likeliness of the extinction of a specialist species by some ecological disturbance too. According to the results for our model, as illustrated in Fig. 8, a series of the extinction of native prey species and the invasion of alien prey species would tend to make the number of available prey species smaller in the apparent competition system, and could make it a 1 prey-1 predator system in which the predator appears as a specialist relying on a specific prey species. Such the apparent exclusion of prey species other than a particular prey species may be referred as “dynamic monophagy” (Holt and Lawton 1994). There are some evidence of the exclusion of phytophargous insect species by shared enemies (see Frank van Veen et al. 2006, and references therein). Even for such a 1 prey-1 predator system as the climax state, it may be possible to have a new prey by its successful invasion, whereas it would hardly occur because the nature of such a successful invader prey species must be rather restricted for the climax state (i.e., with a large value of *r*/*b* in our model). This indicates the resistance of such the climax 1 prey-1 predator system against the alien prey species invasion, while it would be vulnerable to some ecological disturbance for the persistent prey species with respect to the system sustainability as argued in Clavel et al. (2011).

We considered a simple Lotka–Volterra prey-predator system with the per capita growth rate of every prey species given as a general function of its density. The functions for prey species in the system may be different from each other, while they must have the same mathematical features assumed in our modeling section. Our results may change to an extent if some of assumed features of the function is modified, while it would be possible to make the assumptions for the function looser to give the qualitatively same results as obtained in this paper. For example, if we assume a weak Allee effect for the per capita growth rate, it would be the case, as was partially discussed in Holt (1977), whereas it could be regarded still as an open problem because the mathematical arguments corresponding to our results must become rather different and probably very delicate.

On the other hand, naturally for some other types of prey-predator system with predation terms different from the Lotka–Volterra type, stable periodic solution or bistability state can appear as evident in models with switching predation (for example, see Teramoto et al. 1979; Messia et al. 1984; Abrams et al. 1998; Schreiber 2004; Kr̆ivan and Eisner 2006; Serrouya et al. 2015). As implied by Holt (1977); Noy-Meir (1981), even the simple mathematical model of prey-predator population dynamics may show a specific behavior, depending on the assumptions for the dynamical nature of the interaction between prey and predator. Although mathematical works on such nonlinear systems would be interesting and meaningful to give some other insights about the multi species population dynamics and the ecosystem assembly, we do not argue here anymore, but leave the discussion to other past and future related works (Holt 1977; Kr̆ivan 2014; Schreiber and Kr̆ivan 2020).

Although our results are from a simple mathematical model, they could demonstrate that the apparent competition effect could drive some prey species to extinction and contribute to the ecosystem assembly, as indicated by many previous works (for example, Frank van Veen et al. 2006; Bhattarai et al. 2017; Hullé et al. 2022; Lorusso and Faillace 2022). As Holt (2023) discussed, the apparent competition system could be observed in the context other than ecology, for example, in some sociological one. We expect that our mathematical work will be helpful for some theoretical works on some other related problems.

## Data Availability

Not applicable.

## Appendix A Boundedness of solution

There exists the solution of *P* such that $P(t)\equiv 0$ for any *t* according to (1) with $P(0) = 0$. On the other hand, from (1), we can get the following formal equations of the solution:

A1 $$\begin{aligned} H_{i}(t)&= H_i(0)\exp \Big [\displaystyle \int _0^t\big \{ g_i(H_{i}(\tau )) - b_{i} P(\tau )\big \}\, d\tau \Big ] \quad (i = 1, 2, \ldots , n); \end{aligned}$$

A2 $$\begin{aligned} P(t)&= P(0)\exp \Big [-\delta t + \sum _{i = 1}^{n} c_{i} b_{i} \displaystyle \int _0^tH_{i}(\tau )\, d\tau \Big ]. \end{aligned}$$

The formal solution (A2) for *P*(*t*) shows that $P(t) >0$ for any $t>0$ if $P(0) > 0$, because of the uniqueness of the solution for (1).

In the same way, there exists the solution such that $H_i(t)\equiv 0$ for any *t* and any *i* according to (1) with $H_i(0) = 0$. Hence, from the uniqueness of the solution for (1), the formal solution (A1) for $H_i(t)$ shows that $H_i(t) >0$ for any $t>0$ with $H_i (0) >0$. Then, for any $H_i >0$ and $P > 0$, we have

A3 $$\begin{aligned} \left. \displaystyle \frac{dH_i}{dt}\right| _{H_i \ge K_i} = g_i(H_{i})H_i - b_{i} P H_{i} < 0, \end{aligned}$$

because $g_i(H)$ is strictly decreasing in terms of $H>0$ and $g_i(H) \le g_i(K_i) = 0$ for any $H \ge K_i$. Therefore, if $H_i(0)\in (0, K_i]$ ($i=1,2,\dots ,n$), it is impossible that $H_i(t)\ge K_i$ for any $t>0$. Consequently we find that, if $H_i(0) \in (0, K_i]$ and $P(0) > 0$, then $H_i(t) \in (0, K_i)$ at any time $t>0$.

## Appendix B Proof of Theorem 1

By the boundedness of solution shown in Appendix A, we can find that

$$\begin{aligned} \displaystyle \frac{dP}{dt} =\,&\Big (-\delta + \sum _{i = 1}^{n} c_{i} b_{i} H_{i} \Big )P \le \Big (-\delta + \sum _{i = 1}^{n} c_{i} b_{i} K_{i} \Big )P\\ =\,&\delta \Big (-1+\sum _{i=1}^n \frac{c_i b_iK_i}{\delta }\Big )P = \delta \big (-1+{\mathscr {R}}_0^{[n]}\big )P. \end{aligned}$$

Hence from the comparison theorem, we can find that

B4 $$\begin{aligned} P(t) \le P(0)e^{-\delta (1-{\mathscr {R}}_0^{[n]} )t} \end{aligned}$$

for any $t > 0$. Then we find from (B4) that $P(t)\rightarrow 0$ as $t\rightarrow \infty$ if ${\mathscr {R}}_0^{[n]} < 1$.

Next, suppose that $P(t)\rightarrow 0$ as $t\rightarrow \infty$. From (1), we can easily see that $H_i(t)\rightarrow K_i$ ($i=1, 2,\dots , n$) as $P(t)\rightarrow 0$. On the other hand, any equilibrium such that $P=0$ and $H_k = 0$ for some *k* is always unstable because any prey population grows in a monotonic manner independently of the other prey populations when the predator is absent. Thus, if $P(t)\rightarrow 0$, the system (1) asymptotically approaches the equilibrium $(K_1, K_2,\dots , K_n, 0)$. By the local stability analysis for the equilibrium $(K_1, K_2,\dots , K_n, 0)$, we can easily prove that $P(t)\rightarrow 0$ as $t\rightarrow \infty$ only if $\mathscr {R}_0^{[n]} < 1$, while the equilibrium $(K_1, K_2,\dots , K_n, 0)$ is unstable if ${\mathscr {R}}_0^{[n]} > 1$, so that the predator is then persistent.

When ${\mathscr {R}}_0^{[n]} = 1$, we have $\delta =\displaystyle \sum _{i = 1}^nc_ib_iK_i$, which leads to

$$\begin{aligned} \displaystyle \frac{dP}{dt}&= -\sum _{i=1}^n {c_i b_i} (K_i - H_i )P < 0 \end{aligned}$$

for any $t >0$ since $P(t) > 0$ and $H_i(t) <K_i$ for any $t > 0$ (Appendix A). Therefore, $P(t)\rightarrow 0$ as $t\rightarrow 0$ again in this case. These arguments prove Theorem 1.

## Appendix C Proof of Lemma 2

The former equation of (9) shows it necessary for the existence of $E^*_{[k]}$ defined by (8) that $b_iP^*_{[k]}= g_i\big (H_{[k], i}^*\big ) < g_i(0) = r_i$, that is, $P^*_{[k]} < r_i/b_i$ for $i = 1, 2,\dots , k$. This is because $g_i(H)$ is monotonically decreasing in terms of *H* and positive only for $H\in [0, K_i)$. From the numbering of prey species as given by (4), we find that if and only if

C5 $$\begin{aligned} P^*_{[k]} < \dfrac{\ r_k}{\ b_k}, \end{aligned}$$

we have $P^*_{[k]} < r_i/b_i$ for all $i = 1, 2,\dots , k$. This is the proof for the last part of Lemma 2.

The latter equation of (10) determines the predator population size at the equilibrium $E^*_{[k]}$. From the assumptions for $g_i$ given in Sect. 2, the left side of the latter equation of (10), that is, the function ${\mathscr {G}}_k(P^*_{[k]})$ is a strictly decreasing function continuous and differentiable in terms of $P^*_{[k]} > 0$. Thus if the latter equation of (10) has a positive root $P^*_{[k]}$, it must be unique. Then it is necessary for the existence of such a positive root $P^*_{[k]}$ of the latter equation of (10) that ${\mathscr {G}}_k(0) > 1$, that is,

C6 $$\begin{aligned} {\mathscr {G}}_k(0) = \displaystyle \sum _{i=1}^k\dfrac{g_i^{-1}(0)}{K_i}{\mathscr {R}}_{0, i} = \displaystyle \sum _{i=1}^k{\mathscr {R}}_{0, i} = {\mathscr {R}}_0^{[k]} >1, \end{aligned}$$

where ${\mathscr {R}}_0^{[k]}$ is defined in (11) as well as $\mathscr {R}_0^{[n]}$ by (7), and necessarily ${\mathscr {R}}_0^{[k]}\le {\mathscr {R}}_0^{[n]}$ for $k\le n$.

At the same time, from the condition (C5) for the existence of the equilibrium $E^*_{[k]}$, it is necessary for the existence of such a positive root $P^*_{[k]}$ of the latter equation in (10) that ${\mathscr {G}}_k ({r_k}/{b_k} ) < 1$, that is,

C7 $$\begin{aligned} {\mathscr {W}}_k := {\mathscr {G}}_k\big (\frac{\, r_k}{\, b_k}\big ) = \displaystyle \sum _{i=1}^k\dfrac{g_i^{-1}\big (\frac{r_k/b_k}{r_i/b_i}r_i\big )}{K_i}{\mathscr {R}}_{0, i} < 1, \end{aligned}$$

where ${\mathscr {W}}_k$ is defined in (11). Note that conditions (C5) and (C7) are mathematically equivalent to each other.

Now we can find the monotonicity of the sequence $\{{\mathscr {W}}_k\}$:

### Lemma 6

The sequence $\{{\mathscr {W}}_k\}$ is non-decreasing in terms of *k*: ${\mathscr {W}}_1 = 0$, ${\mathscr {W}}_k < {\mathscr {W}}_{k+1}$ if and only if $r_{k+1}/b_{k+1} < r_k/b_k$, and ${\mathscr {W}}_k = \mathscr {W}_{k+1}$ if and only if $r_{k+1}/b_{k+1} = r_k/b_k$.

### Proof

We can easily derive

$$\begin{aligned} {\mathscr {W}}_1&= {\mathscr {G}}_1\big (\frac{\, r_1}{\, b_1}\big ) = \dfrac{g_1^{-1}\big (\frac{r_{1}/b_{1}}{r_1/b_1}r_1\big )}{K_1}{\mathscr {R}}_{0, 1} = \dfrac{g_1^{-1} (r_1 )}{K_1}\mathscr {R}_{0, 1} = \dfrac{0}{K_1}{\mathscr {R}}_{0, 1} = 0. \end{aligned}$$

Next we note the following nature of the function ${\mathscr {G}}_k$:

C8 $$\begin{aligned} {\mathscr {G}}_k\big (\frac{\, r_{k+1}}{\, b_{k+1}}\big )&= \displaystyle \sum _{i=1}^{k}\dfrac{g_i^{-1}\big (\frac{r_{k+1}/b_{k+1}}{r_i/b_i}r_i\big )}{K_i}{\mathscr {R}}_{0, i}\nonumber \\&= \displaystyle \sum _{i=1}^{k+1}\dfrac{g_i^{-1}\big (\frac{r_{k+1}/b_{k+1}}{r_i/b_i}r_i\big )}{K_i}{\mathscr {R}}_{0, i} = {\mathscr {G}}_{k+1}\big (\frac{\, r_{k+1}}{\, b_{k+1}}\big ) = \mathscr {W}_{k+1}, \end{aligned}$$

since $g_i^{-1}\big (\frac{r_{k+1}/b_{k+1}}{r_{k+1}/b_{k+1}}r_i\big )=g_i^{-1}(r_i) = 0$. Then, from the numbering of prey species as given by (4), and the strictly decreasing monotonicity of ${\mathscr {G}}_k$, we have ${\mathscr {G}}_k ({r_{k+1}}/{b_{k+1}} )>{\mathscr {G}}_k ({r_{k}}/{b_{k}} ) = \mathscr {W}_k$ if ${r_{k+1}}/{b_{k+1}} < {r_{k}}/{b_{k}}$, and $\mathscr {G}_k ({r_{k+1}}/{b_{k+1}} )={\mathscr {G}}_k ({r_{k}}/{b_{k}} ) = {\mathscr {W}}_k$ if ${r_{k+1}}/{b_{k+1}} = {r_{k}}/{b_{k}}$. Hence, from (C8), we have the result in Lemma 6. $\square$

In addition, from the strictly decreasing monotonicity of ${\mathscr {G}}_k$ with ${\mathscr {G}}_k(0) = {\mathscr {R}}_0^{[k]}$ shown in (C6), we can find that ${\mathscr {G}}_k ({r_{k+1}}/{b_{k+1}} )<{\mathscr {G}}_k (0)$, that is,

### Lemma 7

$$ {\mathscr {W}}_{k+1} < {\mathscr {R}}_0^{[k]} $$ for every $$k = 1, 2,\dots , n-1$$.

From Lemmas 6 and 7, we have found that ${\mathscr {W}}_k \le {\mathscr {W}}_{k+1} < \mathscr {R}_0^{[k]}$. Consequently, if and only if the conditions (C6) and (C7) are satisfied, the latter equation of (10) has a unique positive root $P^*_{[k]}$ such that the condition (C5) holds, and $H^*_{[k], i}\in (0, K_i)$ ($i=1,2,\dots , k$) is uniquely determined for the equilibrium $E^*_{[k]}$ defined by (8). This result proves Lemma 2.

## Appendix D Proof of Theorem 2

For the equilibrium $E^*_{[n]}$, let us consider the function

D9 $$\begin{aligned} V_{[n]}(t)&:= P^*_{[n]} \Bigg \{\frac{P(t)}{P^*_{[n] }} - 1 - \log \frac{P(t)}{P^*_{[n] }} \Bigg \} + \sum _{i = 1}^{n} c_{i} H^*_{[ n] , i} \Bigg \{\frac{H_{i}(t)}{H^*_{[ n] , i}} - 1 - \log \frac{H_{i}(t)}{H^*_{[n] , i}} \Bigg \}. \end{aligned}$$

From the equations of (1) and the equilibrium values determined by (10) with $k = n$ for $E^*_{[n]}$, we can get the following expression of the derivative of $V_{[n]}(t)$:

D10 $$\begin{aligned} \frac{dV_{[n]}(t)}{dt} = \sum _{i = 1}^{n} c_{i} \{H_{i}(t) - H_{[n], i}^*\} \{g_i\big (H_{i}(t)\big ) - g_i\big (H_{[n], i}^*\big )\}. \end{aligned}$$

Because of the strictly decreasing monotonicity of $g_i$ as assumed in Sect. 2, we have $g_i (H_i) > g_i\big (H_{[n], i}^*\big )$ if and only if $H_i < H_{[n], i}^*$, and $g_i (H_i) < g_i\big (H_{[n], i}^*\big )$ if and only if $H_i > H_{[n], i}^*$. Hence the right side of (D10) is necessarily negative if $H_i \ne H_{[n], i}^*$ about some *i*, that is, $dV_{[n]} / dt < 0$ as long as $H_i \ne H_{[n], i}^*$ about some *i*. Thus we have $dV_{[n]} / dt = 0$ only when $H_i(t) = H_{[n], i}^*$ for every *i*.

Note that $H_i(t)$ cannot remain the value $H_{[n], i}^*$ besides being at the equilibrium $E^*_{[n]}$. Since $H_i(t)$ temporally varies as long as $P(t) \ne P^*_{[n]}$ even when $H_i(t) = H_{[n], i}^*$ for every *i*, we can see that $V_{[n]}(t)$ is monotonically decreasing in terms of $t>0$, even though $dV_{[n]}/dt = 0$ at some moments when $H_i(t) = H_{[n], i}^*$ for every *i* with $P(t) \ne P^*_{[n]}$. Moreover, as long as $P(t) >0$ and $H_i(t)>0$, we have $V_{[n]}\ge 0$, where $V_{[n]}=0$ only at the equilibrium $E^*_{[n]}$.

Therefore we find that $V_{[n]}$ is monotonically decreasing in terms of $t > 0$, positive definite for any $(H_{1}, H_{2},\dots , H_{n}, P)$ other than $E^*_{[n]}$ in ${\mathcal {D}}$ defined by (3), and zero only at the equilibrium $E^*_{[n]}$. This means that the function $V_{[n]}$ is a Lyapunov function for the equilibrium $E^*_{[n]}$, and we can conclude that $E^*_{[n]}$ is globally asymptotically stable in ${\mathcal {D}}$. whenever it exists. Consequently these arguments prove Theorem 2. For the further mathematical information about the analysis on the global stability with Lyapunov function, refer to, for example, Goh (1980); Takeuchi and Adachi (1980); Takeuchi (1996) and the references therein.

## Appendix E Proof of Lemma 3

For the equilibrium $E^*_{[k]}$ defined by (8) with $k < n$, the Jacobi matrix becomes

E11

where $\textrm{diag}_{(j,\ell )}(a_i)$ denotes the $(\ell - j+1)\times (\ell - j+1)$ diagonal matrix which (*k*, *k*)-element is $a_k$ ($k = j, j+1,\dots \ell$), $\text{0 }$ is the zero matrix, and $\Xi _i:= g_{i}(0)-b_iP^*_{[k]}=r_i-b_iP^*_{[k]}$. Then the eigenvalue $\lambda$ is given by the root of the following equation:

E12 $$\begin{aligned} \Psi (\lambda )\prod _{i=k+1}^n\big (\Xi _i - \lambda \big )&=0 \end{aligned}$$

with

$$\begin{aligned} \Psi (\lambda )&= -\lambda \prod _{i=1}^k\Big \{ g_i'(H^*_{[k], i})H^*_{[k], i}-\lambda \Big \}\\&\quad +\sum _{i=1}^k (-1)^{k+i} b_iH^*_{[k], i}\cdot c_ib_iP^*_{[k]}\prod _{j=1,j\ne i}^k \Big \{ g_j'(H^*_{[k], j})H^*_{[k], j}-\lambda \Big \}. \end{aligned}$$

From (E12), $\Xi _i$ ($i=k+1, k+2,\dots , n$) is the eigenvalue, and it is necessary for the local stability of $E^*_{[k]}$ that $\Xi _i \le 0$, that is, $P^*_{[k]}\ge r_i/b_i$ ($i=k+1, k+2,\dots , n$). Because of the numbering of prey species as given by (4), this condition is satisfied if and only if

E13 $$\begin{aligned} P^*_{[k]} \ge \dfrac{\ r_{k+1}}{\ b_{k+1}}. \end{aligned}$$

This proves the latter part of Lemma 3. With the same arguments as in Appendix C for the proof of Lemma 2, we have the following condition equivalent to (E13): $\mathscr {G}_k ({r_{k+1}}/{b_{k+1}} )= {\mathscr {W}}_{k+1}\ge 1$.

From Lemma 7 in Appendix C, if ${\mathscr {W}}_{k+1}\ge 1$, then we have ${\mathscr {R}}_0^{[k]} > 1$. Thus, if ${\mathscr {W}}_k < 1\le {\mathscr {W}}_{k+1}$, we have ${\mathscr {W}}_k< 1< \mathscr {R}_0^{[k]}$, so that $E^*_{[k]}$ exists from Lemma 2. Hence, if the equilibrium $E^*_{[k]}$ exists and is locally asymptotically stable, it is necessary that ${\mathscr {W}}_k < 1\le {\mathscr {W}}_{k+1}$. Note that, if ${\mathscr {W}}_{k+1} < 1$, the equilibrium $E^*_{[k]}$ exists and is unstable, because it holds that $P^*_{[k]} < {r_{k+1}}/{b_{k+1}}$, that is, $\Xi _{k+1} > 0$, which indicates the existence of a positive eigenvalue for the Jacobi matrix (E11).

Next, if the condition ${\mathscr {W}}_k < 1\le {\mathscr {W}}_{k+1}$ is satisfied, the equilibrium $E^*_{[k]}$ exists from Lemma 2, and the condition (E13) holds from the above arguments. We have $\Xi _i\le 0$ ($i=k+1, k+2,\dots , n$) also from the above arguments. Then all diagonal elements of the Jacobi matrix (E11) are non-positive since $g_i'(H^*_{[k], i})H^*_{[k], i}< 0$ with $g_i'(H^*_{[k], i})<0$ and $H^*_{[k], i}>0$. Now we can apply the criterion of asymptotic stability by Jeffries (1974) for the linearized system around the equilibrium $E^*_{[k]}$, represented by the Jacobi matrix (E11). It was applied also by Holt (1977, Appendix I) to prove the locally asymptotic stability of the corresponding equilibrium for the system (1) with prey species growing in the logistic manner as $g_i(H_i) =r_i - \beta _i H_i$ ($i = 1, 2, \dots , n$). The criterion for the Jacobi matrix $\{a_{ij}\}$ consists of five conditions: (i) $a_{ii} \le 0$ for all *i*; (ii) $a_{ij}a_{ji} \le 0$ for all $i\ne j$; (iii) $a_{ij}a_{jk}\cdots a_{\ell q}a_{q i} =0$ for three or more distinct indices; (iv) $\det \{a_{ij}\}\ne 0$; (v) the signed digraph of $\{a_{ij}\}$ “fails” a “color test” defined for a *predation community* by Jeffries (1974) (also refer to Levins 1975; Holt 1977). Since the last condition (v) does not depend on the detail of elements in $\{a_{ij}\}$ but concerns only their signs, the Jacobi matrix (E11) satisfies it as was already found in Holt (1977, Appendix I). It is apparent that the conditions (i) to (iv) hold now for the Jacobi matrix (E11).

Therefore, it is ensured that the equilibrium $E^*_{[k]}$ exists and locally asymptotically stable if the condition ${\mathscr {W}}_k < 1\le {\mathscr {W}}_{k+1}$ is satisfied. In other words, the condition ${\mathscr {W}}_k < 1\le {\mathscr {W}}_{k+1}$ is sufficient for the existence and locally asymptotic stability of $E^*_{[k]}$. Lastly, there arguments prove the former part of Lemma 3.

## Appendix F Proof of Lemma 4

When ${\mathscr {W}}_\ell < 1\le {\mathscr {W}}_{\ell +1}$, any equilibrium $E^*_{[k]}$ defined by (8) with $k > \ell$ does not exist because the existence condition (11) in Lemma 2 cannot be satisfied. Furthermore, even if an equilibrium $E^*_{[k]}$ with some $k < \ell$ exists, it must be unstable. Since ${\mathscr {W}}_k \le \mathscr {W}_{k+1}\le {\mathscr {W}}_\ell$ for any $k < \ell$ because of the non-decreasing monotonicity of the sequence $\{{\mathscr {W}}_k\}$ as shown in Lemma 6 of Appendix C, we have ${\mathscr {W}}_k \le {\mathscr {W}}_{k+1} < 1$ for $k < \ell$ with ${\mathscr {W}}_\ell < 1$. Therefore the equilibrium $E^*_{[k]}$ with $k < \ell$ is unstable even if it exists, since there is some positive eigenvalue for the Jacobian matrix.

On the other hand, we may consider the other type of equilibrium different from that defined by (8), for example, like

F14 $$\begin{aligned} (H_{1}, H_{2},\dots , H_{n}, P)&= (H^*_{[k'] , 1},\dots , H^*_{[k'] , \ell -1},0,H^*_{[k'] , \ell +1},\nonumber \\&\qquad \dots , H^*_{[k'] , k'+1}, \underbrace{0,\dots , 0}_{n-k'}, P^*_{[k']}) \end{aligned}$$

with $H^*_{[k'], i}> 0$ for $i = 1,\dots , \ell -1, \ell +1,\dots , k'+1$ and $P^*_{[k']} > 0$. With the same arguments as the proof of Lemma 2 in Appendix C, we can easily find it necessary for the existence of the equilibrium (F14) that $P^*_{[k']}<r_{k'}/b_{k'}$. Moreover with the same arguments as in Appendix E, we can find a necessary condition for the local stability of the equilibrium (F14) that $P^*_{[k']}\ge r_{\ell }/b_{\ell }$. From the numbering of prey species as given by (4), it is impossible to satisfy these necessary conditions for the existence and local stability of equilibrium (F14) at the same time. Thus, even if the equilibrium (F14) exists, it must be necessarily unstable. By the same arguments, we can prove that any equilibrium other than (8) must be unstable even if it exists. Consequently we find that only the type of equilibrium defined by (8) can be locally asymptotically stable.

Further from Theorem 2, we have noted that the equilibrium $E^*_{[n]}$ exists and is globally asymptotically stable if ${\mathscr {W}}_n< 1 <{\mathscr {R}}_0^{[n]}$. Indeed, when ${\mathscr {W}}_n< 1 <{\mathscr {R}}_0^{[n]}$, any other existing equilibrium $E^*_{[k]}$ with $k < n$ is unstable since it cannot hold that ${\mathscr {W}}_k < 1 \le {\mathscr {W}}_{k+1}$ in the case, because of the non-decreasing monotonicity of the sequence $\{{\mathscr {W}}_k\}$. Inversely when ${\mathscr {W}}_k < 1 \le \mathscr {W}_{k+1}$ for a $k < n$, the equilibrium $E^*_{[n]}$ does not exist since it cannot hold that ${\mathscr {W}}_n< 1 <{\mathscr {R}}_0^{[n]}$. Accordingly with these arguments, we can conclude the result given as Lemma 4.

## Appendix G Proof of Theorem 3

From Theorem 1, if $\mathscr {R}_0^{[n]} \le 1$, the predator goes extinct while all preys persist at the equilibrium for the system (1). Then the equilibrium $E^*_{[k]}$ with $P^*_{[k]}>0$ is not defined. The definition of the specific number *s* given by (12) is valid only when the predator persists with ${\mathscr {R}}_0^{[n]}>1$. As long as arguing the stability of the equilibrium $E^*_{[k]}$, it is necessary and satisfactory to consider the case where ${\mathscr {R}}_0^{[n]} > 1$.

The sequence $\{{\mathscr {W}}_k\}$ is non-decreasing in terms of *k* as shown by Lemma 6 in Appendix C, and satisfies that ${\mathscr {W}}_{k+1} < {\mathscr {R}}_0^{[k]}$ for every $k = 1, 2,\dots , n-1$ as shown by Lemma 7 in Appendix C. Lemma 4 indicates that, if there is a locally asymptotically stable equilibrium, then it is *unique*. Hence, from Lemma 4, when the locally asymptotically stable equilibrium is given by $E^*_{[s]}$ with $s < n$, it is satisfied that ${\mathscr {W}}_s< 1 \le \mathscr {W}_{s+1} < {\mathscr {R}}_0^{[s]}$. Then, for any unstable equilibrium $E^*_{[k]}$ with $k > s$, it cannot hold that ${\mathscr {W}}_k < 1$, since ${\mathscr {W}}_k \ge {\mathscr {W}}_{s+1} \ge 1$ from the non-decreasing monotonicity of the sequence $\{{\mathscr {W}}_k\}$. In contrast, for any unstable equilibrium $E^*_{[k]}$ with $k < s$, it holds that ${\mathscr {W}}_k < 1$, since ${\mathscr {W}}_k \le \mathscr {W}_{s} < 1$. Moreover, even for such a $k < s$, it may hold that ${\mathscr {W}}_k < 1\le {\mathscr {R}}_0^{[k]}$. Therefore, these arguments show that the order *s* for the locally asymptotically stable equilibrium $E^*_{[s]}$ is uniquely determined by (12).

In the case of $s = n$, we already have the result of Theorem 2 with Lemma 2. When exists the locally asymptotically stable equilibrium $E^*_{[s]}$ with $s < n$ defined by (12), let us consider the function

G15 $$\begin{aligned} V_{[s]}(t) :&= P^*_{[s]} \bigg \{\frac{P(t)}{P^*_{[s]}} - 1 - \log \frac{P(t)}{P^*_{[s]}} \bigg \} \nonumber \\&\quad + \sum _{i = 1}^{s} c_{i} H^*_{[ s] , i} \bigg \{\frac{H_{i}(t)}{H^*_{[ s] , i}} - 1 - \log \frac{H_{i}(t)}{H^*_{[ s] , i}} \bigg \} + \sum _{i=s+1}^nc_iH_i(t). \end{aligned}$$

From the equations of (1) and the equilibrium values determined by (10) with $k = s$ for $E^*_{[s]}$, we can derive the following expression of the derivative of $V_{[s]}(t)$:

G16 $$\begin{aligned} \frac{dV_{[s]}(t)}{dt}&= \sum _{i = 1}^{s} c_{i} \{H_{i}(t) - H_{[s], i}^*\} \big \{g_i\big (H_{i}(t)\big ) - g_i\big (H_{[s], i}^*\big )\big \} \nonumber \\&\quad +\sum _{i={s}+1}^n c_i H_i(t)\big \{ g_i\big (H_{i}(t)\big ) - b_{i} P^*_{[s]}\big \}, \end{aligned}$$

The first term is necessarily negative as long as $H_i \ne H_{[s], i}^*$ about some $i\in \{1, 2,\dots , s\}$, which can be proven by the same arguments as the proof of Theorem 2 in Appendix D. As shown in Lemmas 2 and 3, we have $P^*_{[s]} \in [ r_{s+1}/b_{s+1}, r_s/b_s)$ for the locally asymptotically stable equilibrium $E^*_{[s]}$. Then we can find that

$$\begin{aligned} g_i\big (H_{i}(t)\big ) - b_{i} P^*_{[s]}&\le g_i\big (H_{i}(t)\big ) - b_{i}\,\frac{\, r_{s+1}}{\, b_{s+1}} = g_i\big (H_{i}(t)\big ) - \frac{r_{s+1}/b_{s+1}}{r_i/b_i}\, r_i\\&\le g_i\big (H_{i}(t)\big ) - r_i = g_i\big (H_{i}(t)\big ) - g_i(0) < 0, \end{aligned}$$

for any $i \ge s+1$ because ${r_{s+1}/b_{s+1}}\ge {r_i/b_i}$ for any $i \ge s+1$ by the numbering of prey species as given by (4), and $g_i (0) = r_i > g_i(H_i)$ for any $H_i >0$, following the strictly decreasing monotonicity of $g_i$ as assumed in Sect. 2. Hence we have found that $dV_{[s]}(t)/dt < 0$ in ${\mathcal {D}}\backslash E^*_{[s]}$.

It can be easily found from (G15) and (G16) that $V_{[s]}$ and $dV_{[s]}/dt$ are zero only at the equilibrium $E^*_{[s]}$, and further that $V_{[s]}$ is positive definite for any $(H_{1}, H_{2},\dots , H_{n}, P)$ other than $E^*_{[s]}$ in ${\mathcal {D}}$ defined by (3) in Lemma 1. This means that the function $V_{[s]}$ is a Lyapunov function for the equilibrium $E^*_{[s]}$ about the solution of the system (1), and we can conclude that $E^*_{[s]}$ is globally asymptotically stable in ${\mathcal {D}}$ when it is locally asymptotically stable. Consequently these arguments prove Theorem 3.

## Appendix H Proof of Theorem 4

Let us suppose the extermination of prey species *k* from the equilibrium $E^*_{[n]}$. From Theorem 3, the coexistent equilibrium $E^*_{[n\backslash k]}$of the predator and $n-1$ rest prey species is globally asymptotically stable if and only if $\mathscr {W}_{n\backslash k}< 1 <{\mathscr {R}}_0^{[n\backslash k]}$, where

H17 $$\begin{aligned} {\mathscr {W}}_{n \backslash k} := {\mathscr {W}}_n- \dfrac{g_k^{-1}\big (\frac{r_n/b_n}{r_k/b_k}r_k\big )}{K_k}{\mathscr {R}}_{0, k} ;\quad {\mathscr {R}}_0^{[n\backslash k]} := {\mathscr {R}}_0^{[n]}-{\mathscr {R}}_{0, k}. \end{aligned}$$

From Theorem 1, if and only if ${\mathscr {R}}_0^{[n\backslash k]}\le 1$, the predator goes extinct at the system with $n-1$ available prey species after the extermination of prey species *k* from $E^*_{[n]}$. Otherwise it persists. From the asymptotic stability of $E^*_{[n]}$, it is supposed now that ${\mathscr {W}}_{n}< 1 <{\mathscr {R}}_0^{[n]}$. Then, from (H17) we have

$$\begin{aligned} {\mathscr {W}}_{n \backslash k} < 1- \dfrac{g_k^{-1}\big (\frac{r_n/b_n}{r_k/b_k}r_k\big )}{K_k}{\mathscr {R}}_{0, k} ;\quad {\mathscr {R}}_0^{[n\backslash k]} > 1-{\mathscr {R}}_{0, k}. \end{aligned}$$

The former inequality shows that ${\mathscr {W}}_{n \backslash k} < 1$. Hence, when ${\mathscr {R}}_0^{[n\backslash k]}> 1$, it is satisfied that ${\mathscr {W}}_{n\backslash k}< 1 <{\mathscr {R}}_0^{[n\backslash k]}$. These arguments prove Theorem 4.

## Appendix I Proof of Theorem 5

Let us consider the invasion of an alien prey species with its parameters $c_\bullet$, $b_\bullet$, and function $g_\bullet$ accompanied with $r_\bullet$ and $K_\bullet$. We now define the following ${\mathscr {R}}_0^{[n \oplus 1]}$ and ${\mathscr {W}}_{n \oplus 1}$ for the system invaded by such an alien prey species:

$$\begin{aligned} {\mathscr {R}}_0^{[n \oplus 1]}&:= {\mathscr {R}}_0^{[n]}+\mathscr {R}_{0,\bullet };\\ {\mathscr {W}}_{n \oplus 1}&:= \left\{ \begin{array}{cl} {\mathscr {W}}_n+\dfrac{g_\bullet ^{-1}\big (\frac{r_n/b_n}{r_\bullet /b_\bullet }r_\bullet \big )}{K_\bullet }{\mathscr {R}}_{0, \bullet } & \text{ if } \dfrac{r_\bullet }{b_\bullet }\ge \dfrac{r_n}{b_n} ; \\ {\mathscr {G}}_n\big (\dfrac{\, r_\bullet }{\, b_\bullet }\big ) = \displaystyle \sum _{i=1}^n\dfrac{g_i^{-1}\big (\frac{r_\bullet /b_\bullet }{r_i/b_i}r_i\big )}{K_i}{\mathscr {R}}_{0, i} & \text{ if } \dfrac{r_\bullet }{b_\bullet }< \dfrac{r_n}{b_n} \end{array} \right. \end{aligned}$$

with ${\mathscr {R}}_{0, \bullet }:=c_\bullet b_\bullet K_\bullet /\delta$. Note that ${\mathscr {G}}_n ({r_\bullet }/{b_\bullet } ) >{\mathscr {G}}_n ({r_n}/{b_n} ) ={\mathscr {W}}_n$ for $r_\bullet /b_\bullet < r_n/b_n$, since ${\mathscr {G}}_n (x)$ is a strictly decreasing function in terms of $x > 0$.

Theorems 2 and 3 say that the system transfers to the coexistent equilibrium $E^*_{[n\oplus 1]}$ of the predator, all native prey species, and the alien prey species if and only if ${\mathscr {W}}_{n \oplus 1}< 1 < {\mathscr {R}}_0^{[n \oplus 1]}$. Since necessarily ${\mathscr {R}}_0^{[n \oplus 1]}> 1$ when $\mathscr {R}_0^{[n]} >1$, Theorem 1 shows that the predator must persist with the invasion of any alien prey species. Then we can get the following results:

### Lemma 8

When ${\mathscr {W}}_{n}< 1 < {\mathscr {R}}_0^{[n]}$, the coexistent equilibrium $E^*_{[n\oplus 1]}$ of the predator, all native prey species, and the alien prey species is globally asymptotically stable if and only if the condition (15) or (16) holds. Otherwise some native species or the alien prey species goes extinct.

From Corollary 3 in Sect. 7, if ${r_\bullet }/{b_\bullet }={r_n}/{b_n}$, no extinction of native prey species occurs even after the invasion of the alien prey species because ${\mathscr {W}}_{n \oplus 1} = {\mathscr {W}}_n$. This is included in the condition (16).

Since we suppose the asymptotically stable equilibrium $E^*_{[n]}$ of the system with ${\mathscr {W}}_{n}< 1 < {\mathscr {R}}_0^{[n]}$ before the invasion of the alien prey species, we find the following result of a possible return of the system to the equilibrium $E^*_{[n]}$, making use of Lemma 3 and Theorem 3:

### Lemma 9

When ${\mathscr {W}}_{n}< 1 < {\mathscr {R}}_0^{[n]}$, the system returns to $E^*_{[n]}$ with the extinction of alien prey species if the condition (13) is satisfied.

The condition (13) is complementary to the condition (15). Therefore the extinction of some native species by the invasion of an alien prey species could occur only if ${r_\bullet }/{b_\bullet }>{r_n}/{b_n}$.

Since Lemma 3 shows that only an equilibrium of the type given by (8) can be asymptotically stable, if the alien prey species goes extinct with ${r_\bullet }/{b_\bullet }>{r_n}/{b_n}$, then so do all native prey species *k* with ${r_k}/{b_k}\le {r_\bullet }/{b_\bullet }$. Then, again from Lemma 3, any equilibrium $E^*_{[\ell ]}$ for $\ell$ with ${r_\ell }/{b_\ell }> {r_{\ell +1}}/{b_{\ell +1}}>{r_\bullet }/{b_\bullet }$ cannot be asymptotically stable. This is because the asymptotic stability of such $E^*_{[\ell ]}$ requires the condition that ${\mathscr {W}}_{\ell } < 1 \le {\mathscr {W}}_{\ell + 1}$, which cannot be satisfied since ${\mathscr {W}}_{\ell + 1}< {\mathscr {W}}_{n}< 1 < {\mathscr {R}}_0^{[n]}$ by the increasing monotonicity of the sequence $\{{\mathscr {W}}_k\}$. Hence the equilibrium $E^*_{[\ell ]}$ with the extinction of the alien prey species must satisfy that ${r_\ell }/{b_\ell }> {r_\bullet }/{b_\bullet }\ge {r_{\ell +1}}/{b_{\ell +1}}$ and

$$\begin{aligned} {\mathscr {W}}_\ell < 1 \le {\mathscr {W}}_{\ell \oplus 1} := \mathscr {G}_\ell \big (\frac{\, r_\bullet }{\, b_\bullet }\big ) = \displaystyle \sum _{i=1}^\ell \dfrac{g_i^{-1}\big (\frac{r_\bullet /b_\bullet }{r_i/b_i}r_i\big )}{K_i}{\mathscr {R}}_{0,i}, \end{aligned}$$

if it could exist with the asymptotic stability. However, from the strictly decreasing monotonicity of the function ${\mathscr {G}}_\ell$ and (C8) shown in Appendix C, we find that

$$\begin{aligned} {\mathscr {W}}_{\ell \oplus 1} ={\mathscr {G}}_\ell \big (\frac{\, r_\bullet }{\, b_\bullet }\big ) \le {\mathscr {G}}_\ell \big (\frac{\, r_{\ell +1}}{\, b_{\ell + 1}}\big )&= {\mathscr {G}}_{\ell +1}\big (\frac{\, r_{\ell +1}}{\, b_{\ell + 1}}\big ) = {\mathscr {W}}_{\ell + 1}<{\mathscr {W}}_n < 1. \end{aligned}$$

Therefore such the equilibrium $E^*_{[\ell ]}$ cannot be asymptotically stable even if it exists. As a result, we obtain the following result:

### Lemma 10

When ${\mathscr {W}}_{n}< 1 < {\mathscr {R}}_0^{[n]}$, by the invasion of an alien prey species with ${r_\bullet }/{b_\bullet }>{r_n}/{b_n}$, the system transfers to an equilibrium at which the alien prey species necessarily persists.

From Lemma 3 and Theorem 3, the system transfers to an asymptotically stable equilibrium $E^*_{[\ell \oplus 1]}$ with $r_\ell /b_\ell \ge {r_\bullet }/{b_\bullet }> r_{\ell +1}/b_{\ell +1}$, if and only if ${\mathscr {W}}_{\ell \oplus 1}<1<\mathscr {R}_0^{[\ell \oplus 1]} = {\mathscr {R}}_0^{[\ell ]} +{\mathscr {R}}_{0, \bullet }$ and

$$\begin{aligned} {\mathscr {W}}_{\ell + 1\oplus 1}&:= {\mathscr {W}}_{\ell +1} + \dfrac{g_\bullet ^{-1}\big (\frac{r_{\ell + 1}/b_{\ell + 1}}{r_\bullet /b_\bullet }r_\bullet \big )}{K_\bullet }\mathscr {R}_{0, \bullet } \ge 1, \end{aligned}$$

that is, if and only if

I18 $$\begin{aligned} 1-\dfrac{g_\bullet ^{-1}\big (\frac{r_{\ell + 1}/b_{\ell + 1}}{r_\bullet /b_\bullet }r_\bullet \big )}{K_\bullet }\mathscr {R}_{0, \bullet } \le {\mathscr {W}}_{\ell +1}, \end{aligned}$$

where we used ${\mathscr {W}}_{\ell + 1\oplus 1} < \mathscr {R}_0^{[\ell \oplus 1]}$ and ${\mathscr {W}}_{\ell \oplus 1} < 1$ which always hold as shown in Appendix C and in the above. The number of extinct native prey species at the equilibrium $E^*_{[\ell \oplus 1]}$ is $n-\ell$. In this case with the condition (I18), the number of extinct native prey species is maximum at the newly established equilibrium state by the invasion of such an alien prey species.

Especially in an extremal case with $r_\bullet /b_\bullet > {r_1}/{b_1}$, all native prey species become extinct at the newly established equilibrium state by the invasion of such an alien prey species if and only if the condition (I18) is satisfied formally for $\ell = 0$, that is, if and only if

I19 $$\begin{aligned} 1-\dfrac{g_\bullet ^{-1}\big (\frac{r_{1}/b_{1}}{r_\bullet /b_\bullet }r_\bullet \big )}{K_\bullet }{\mathscr {R}}_{0, \bullet } \le \mathscr {W}_1 = 0, \end{aligned}$$

which gives the condition (15). In the other extremal case with $r_{n-1}/b_{n-1} \ge {r_\bullet }/{b_\bullet }>r_n/b_n$, the equilibrium $E^*_{[n-1\oplus 1]}$ becomes asymptotically stable if and only if $\mathscr {W}_{n-1\oplus 1}<1\le {\mathscr {W}}_{n\oplus 1}$, that is, if and only if

I20 $$\begin{aligned} 1-\dfrac{g_\bullet ^{-1}\big (\frac{r_{n}/b_{n}}{r_\bullet /b_\bullet }r_\bullet \big )}{K_\bullet }{\mathscr {R}}_{0, \bullet } \le \mathscr {W}_{n}, \end{aligned}$$

where we used ${\mathscr {W}}_{n-1\oplus 1}\le {\mathscr {W}}_n < 1$ as shown in the above with respect to the feature that $\mathscr {W}_{\ell \oplus 1}\le {\mathscr {W}}_{\ell +1}$. In the case with $r_{n-1}/b_{n-1} \ge {r_\bullet }/{b_\bullet }>r_n/b_n$, if and only if the alien prey species satisfies the condition (I20), only the prey species *n* becomes extinct at the newly established equilibrium. Otherwise with such an alien species, no extinction of prey species occurs even at the newly established equilibrium, which has been already indicated by the condition (16).

By the same arguments, we can consider the condition with which the system transfers to the asymptotically stable equilibrium $E^*_{[k^*\oplus 1]}$ by the invasion of an alien prey species with ${r_\bullet }/{b_\bullet }>r_{k^*}/b_{k^*}\ge r_n/b_n$. From Lemma 3 and Theorem 3, such a state transition occurs if and only if ${\mathscr {W}}_{k^*\oplus 1}<1\le \mathscr {W}_{k^*+1\oplus 1}$. Since we have

$$\begin{aligned} {\mathscr {W}}_{k^*\oplus 1} = {\mathscr {W}}_{k^*} + \dfrac{g_\bullet ^{-1}\big (\frac{r_{k^*}/b_{k^*}}{r_\bullet /b_\bullet }r_\bullet \big )}{K_\bullet }{\mathscr {R}}_{0, \bullet } ;\quad {\mathscr {W}}_{k^*+1\oplus 1} = {\mathscr {W}}_{k^*+1} + \dfrac{g_\bullet ^{-1}\big (\frac{r_{k^*+1}/b_{k^*+1}}{r_\bullet /b_\bullet }r_\bullet \big )}{K_\bullet }{\mathscr {R}}_{0, \bullet } \end{aligned}$$

with $r_{k^*}/b_{k^*}< {r_\bullet }/{b_\bullet }$, the necessary and sufficient condition becomes

I21 $$\begin{aligned} {\mathscr {W}}_{k^*} < 1-\dfrac{g_\bullet ^{-1}\big (\frac{r_{k^*}/b_{k^*}}{r_\bullet /b_\bullet }r_\bullet \big )}{K_\bullet }{\mathscr {R}}_{0, \bullet } \quad \text{ and }\quad 1-\dfrac{g_\bullet ^{-1}\big (\frac{r_{k^*+1}/b_{k^*+1}}{r_\bullet /b_\bullet }r_\bullet \big )}{K_\bullet }{\mathscr {R}}_{0, \bullet } \le {\mathscr {W}}_{k^*+1}. \end{aligned}$$

By the alien prey species satisfying the condition (I21) with ${r_\bullet }/{b_\bullet }>r_n/b_n$, the number of extinct native prey species at the newly established equilibrium becomes $n-k^*$ after the state transition.

From the decreasing monotonicity of $g^{-1}$ and the increasing monotonicity of the sequence $\{{\mathscr {W}}_k\}$, we now note that the condition (I21) is equivalent to the following:

I22 $$\begin{aligned} \begin{aligned}&1-\dfrac{g_\bullet ^{-1}\big (\frac{r_{j}/b_{j}}{r_\bullet /b_\bullet }r_\bullet \big )}{K_\bullet }{\mathscr {R}}_{0, \bullet } >{\mathscr {W}}_{j} \quad \text{ for } \text{ all }\ j=\ell +1, \dots , k^*;\\&\text{ and }\\&1-\dfrac{g_\bullet ^{-1}\big (\frac{r_{j}/b_{j}}{r_\bullet /b_\bullet }r_\bullet \big )}{K_\bullet }{\mathscr {R}}_{0, \bullet } \le {\mathscr {W}}_{j} \quad \text{ for } \text{ all }\ j=k^* +1, \dots , n. \end{aligned} \end{aligned}$$

Hence every native prey species *j* satisfying the latter condition of (I22) is extinct at the newly established equilibrium by the alien prey species with ${r_\bullet }/{b_\bullet }>r_n/b_n$.

We note that the condition (I22) is applicable also when $r_{n-1}/b_{n-1} \ge {r_\bullet }/{b_\bullet }>r_n/b_n$, as indicated by the result given by (I20). Besides, if no prey species *j* satisfies the latter condition of (I22), then no native prey species becomes extinct at the newly established equilibrium. In such a case, we find that the former condition of (I22) with $j = n$ coincides with the condition (16). Actually, if the condition (16) is satisfied, all native species *j* satisfy the former condition of (I22) because of the decreasing monotonicity of $g^{-1}$ and the increasing monotonicity of the sequence $\{{\mathscr {W}}_k\}$.

Consequently from those arguments, we can get the following result on an asymptotically stable equilibrium newly established by the invasion of an alien prey species with ${r_\bullet }/{b_\bullet }>r_n/b_n$:

### Lemma 11

After an alien prey species with ${r_\bullet }/{b_\bullet }>{r_n}/{b_n}$ invades in the system (1) with ${\mathscr {W}}_{n}< 1 < {\mathscr {R}}_0^{[n]}$, the system transfer to an equilibrium at which every native prey species $\ell$ satisfying the condition (14) is extinct. If no native prey species $\ell$ satisfies the condition (14), the system transfers to the coexistent equilibrium of the predator, all native prey species, and the alien prey species.

Finally from these results of Lemmas 8–11, we obtain the result of Theorem 5.

## Appendix J Proof of Theorem 6

If the predator goes extinct after the extermination of a prey species *k* at the asymptotically stable coexistent equilibrium $E_{[n]}^*$ of (1), then the theorem holds. Thus, from Theorem 4, we hereafter consider the case where the system transfers to the equilibrium $E_{[n\backslash k]}^*$ with the persistent predator and the remained $n-1$ prey species after the extermination of a prey species *k*. Then assume that ${\mathscr {W}}_n< 1 <{\mathscr {R}}_0^{[n]}$ and ${\mathscr {W}}_{n\backslash k}< 1 <{\mathscr {R}}_0^{[n\backslash k]}$.

First we prove the following feature of the equilibrium predator population size $P^*_{[n\backslash k]}$ at $E_{[n\backslash k]}^*$:

### Lemma 12

$$ P^*_{[n\backslash k]}< r_k/b_k $$.

### Proof

Suppose that $P^*_{[n\backslash k]}\ge r_k/b_k$ with $k < n$. Then, from the former equation of (9), we have

J23 $$\begin{aligned} g_i\big (H_{[n\backslash k] , i}^*\big ) = b_iP^*_{[n\backslash k]} \ge b_i\frac{\, r_k}{\, b_k} = \frac{r_k/b_k}{r_i/b_i}r_i \ge r_i \end{aligned}$$

for $i > k$, because of the numbering of prey species as given by (4). On the other hand, since $H_{[n\backslash k], i}^* > 0$ and $g_i\big (H_{[n\backslash k], i}^*\big ) = b_iP^*_{[n\backslash k]} > 0$ for $i > k$ from the existence of $E_{[n\backslash k]}^*$, it must hold that $g_i\big (H_{[n\backslash k], i}^*\big ) < g_i (0) = r_i$, because of the strictly decreasing monotonicity of $g_i$. This indicates that the inequality (J23) is contradictory to the existence of $P^*_{[n\backslash k]} > 0$. Therefore, it does not hold that $P^*_{[n\backslash k]}\ge r_k/b_k$ with $k < n$. Hence this lemma holds for $k < n$. When prey species *n* is exterminated, that is, when $k = n$, we have $P^*_{[n\backslash k]} = P^*_{[n - 1]}< r_{n-1}/b_{n-1}$ from Lemma 2, which matches this lemma. Consequently this lemma has been proven. $\square$

Next, from the latter equation of (10), the equilibrium predator population sizes $P^*_{[n]}$ and $P^*_{[n\backslash k]}$ at $E_{[n]}^*$ and $E_{[n\backslash k]}^*$ are given by the root of following equations respectively:

J24 $$\begin{aligned} {\mathscr {G}}_n(P^*_{[n]})=\sum _{i=1}^n\dfrac{g_i^{-1}\big (b_iP^*_{[n]}\big )}{K_i}{\mathscr {R}}_{0, i}=1 ;\quad \sum _{i=1,\, \ne k}^n\dfrac{g_i^{-1}\big (b_iP^*_{[n\backslash k]}\big )}{K_i}{\mathscr {R}}_{0, i}=1. \end{aligned}$$

The second equation can be rewritten with the function $\mathscr {G}_n$ as

J25 $$\begin{aligned} {\mathscr {G}}_n(P^*_{[n\backslash k]}) = 1+\dfrac{g_k^{-1}\big (b_kP^*_{[n\backslash k]}\big )}{K_k}{\mathscr {R}}_{0, k}. \end{aligned}$$

Then from Lemma 12, we have

$$\begin{aligned} \dfrac{g_k^{-1}\big (b_kP^*_{[n\backslash k]}\big )}{K_k}\mathscr {R}_{0, k} > \dfrac{g_k^{-1}\big (b_k\cdot r_k/b_k\big )}{K_k}{\mathscr {R}}_{0, k} = \dfrac{g_k^{-1}(r_k)}{K_k}{\mathscr {R}}_{0, k} =0 \end{aligned}$$

because of the strictly decreasing monotonicity of $g^{-1}$. Thus, from (J25), we find that ${\mathscr {G}}(P^*_{[n\backslash k]}) > 1$. Since the function ${\mathscr {G}}(P)$ is strictly decreasing, continuous, and differentiable in terms of $P>0$, the root of ${\mathscr {G}}(P) = 1$ is greater than that of ${\mathscr {G}}(P) = q > 1$. Thus, from the above equations (J24) and (J25) to determine $P^*_{[n]}$ and $P^*_{[n\backslash k]}$, we must have $P^*_{[n\backslash k]}<P^*_{[n]}$. Lastly these arguments prove Theorem 6.

## Appendix K Proof of Theorem 7

Since the result of Corollary 5 has shown the result of Theorem 7 in the case where the successful invasion of an alien prey species does not cause the extinction of any native prey species, it is sufficient to prove the theorem for the case where the successful invasion of an alien prey species induces the extinction of some native prey species. From Theorem 5, we consider the case where $r_\bullet /b_\bullet > r_n/b_n$, since the invasion of an alien prey species can cause the extinction of some native prey species.

Now suppose that every native prey species $\ell > k^*$ become extinct at the newly established equilibrium state $E^*_{[k^*\oplus 1]}$, when $n-k^*$ native prey species go extinct by the invasion of the alien prey species. Firstly let us consider the case where $r_{k^*}/b_{k^*} < r_\bullet /b_\bullet$. From Corollary 4, we have $P^*_{[n]}<r_{n}/b_{n}$ and $P^*_{[k^*\oplus 1]}\ge r_{k^*+1}/b_{k^*+1}$ for $k^* < n$, where $r_{n}/b_{n}\le r_{k^*+1}/b_{k^*+1}< r_{k^*}/b_{k^*} < r_\bullet /b_\bullet$ since the asymptotically stable equilibrium $E^*_{[k^*\oplus 1]}$ with $k^* < n$ requires the condition that $r_{k^*+1}/b_{k^*+1} \ne r_{k^*}/b_{k^*}$ from Corollary 3. Therefore, we find that $P^*_{[n]}<r_{n}/b_{n}\le r_{k^*+1}/b_{k^*+1}\le P^*_{[k^*\oplus 1]}$. Secondly let us consider the case where $r_{k^*}/b_{k^*} \ge r_\bullet /b_\bullet > r_{k^*+1}/b_{k^*+1}\ge r_n/b_n$. Also in this case, the same arguments result in the same condition. Hence these arguments prove the result in Theorem 7 when some native prey species is extinct at the newly established equilibrium $E^*_{[k^*\oplus 1]}$. As a result, we have proven the first part of Theorem 7.

By a similar argument, we can prove the second part of Theorem 7. Let us consider two distinctive cases where $r_\bullet /b_\bullet > r_n/b_n$ and $r_\blacklozenge /b_\blacklozenge > r_n/b_n$, when the invasion of an alien prey species causes the extinction of some native prey species in both cases. Assume that every native prey species $\ell > k^*$ become extinct by the invasion of an alien prey species with $r_\bullet /b_\bullet > r_n/b_n$, while every native prey species $\ell >k^{\dagger }$ become extinct by that of another alien prey species with $r_\blacklozenge /b_\blacklozenge > r_n/b_n$, where the latter case induces the larger number of the extinction of native prey species, that is, $k^{\dagger } < k^*$. From the numbering for the native prey species by (4) and Corollary 3, we simultaneously assume that $r_{k^*}/b_{k^*}< r_{k^{\dagger }}/b_{k^{\dagger }}$ as the mathematical consistency in our modeling. Now from Corollary 4, we have $P^*_{[k^*\oplus 1]}< r_{k^*}/b_{k^*}$ at the newly established equilibrium $E^*_{[k^*\oplus 1]}$ by the invasion of the former alien prey species, and $P^*_{[k^{\dagger }\oplus 1]}\ge r_{k^{\dagger }}/b_{k^{\dagger }}$ at the newly established equilibrium $E^*_{[k^{\dagger }\oplus 1]}$ by the invasion of the latter alien prey species. Therefore we find that $P^*_{[k^*\oplus 1]}< r_{k^*}/b_{k^*}< r_{k^{\dagger }}/b_{k^{\dagger }} \le P^*_{[k^{\dagger }\oplus 1]}$. Finally this condition proves the second part of Theorem 7.

## Appendix L Proof of Corollary 5

The invasion of an alien prey species satisfying the condition (14) in Theorem 5 for $\ell = 1$ leads the system to the equilibrium $E^*_{[\bullet ]}$. In contrast, we note that sufficiently small $b_\bullet$ must cause no extinction of any native prey species in the system (1). The condition (16) for such a coexistent equilibrium when ${r_\bullet }/{b_\bullet }>{r_n}/{b_n}$ is rewritten as

L26 $$\begin{aligned} \dfrac{\ c_\bullet }{\delta }\, b_\bullet g_\bullet ^{-1}\big (\frac{r_{n}}{b_{n}}b_\bullet \big ) <1-{\mathscr {W}}_{n}. \end{aligned}$$

Since the function $h_\bullet (x):= xg^{-1}_\bullet (x)$ is continuous for $x>0$ with $h_\bullet (0) = 0$, the condition (L26), that is, (16) is satisfied for sufficiently small $b_\bullet$. An alien prey population with such a predation rate cannot contribute to the growth rate of predator population, so that the alien prey population does not affect the persistence of any native prey species by the apparent competition effect. This feature of the $b_\bullet$-dependence on the persistence of native prey species is clearly seen in the numerical result of Fig. 6. Hence the range of $b_\bullet$ to have the asymptotically stable $E^*_{[\bullet ]}$ must have a lower bound, $b_\bullet = b_\bullet ^-$, at which the asymptotically stable equilibrium switches to $E^*_{[1\oplus \bullet ]}$ with only native prey species 1, the alien prey species, and the predator.

Further it is clear that there is a upper bound for the range of $b_\bullet$, $b_\bullet = b_\bullet ^+$, to have the asymptotically stable $E^*_{[\bullet ]}$, since the condition (14) must be satisfied with ${r_\bullet }/{b_\bullet }>{r_1}/{b_1}$. Also at the upper bound $b_\bullet = b_\bullet ^+$, the asymptotically stable equilibrium switches to $E^*_{[1\oplus \bullet ]}$. The stability switch to $E^*_{[1\oplus \bullet ]}$ at the lower and upper bounds follows the continuity of the condition (14) in terms of $b_\bullet$.

Now from (10), we have

$$\begin{aligned} P^*_{[\bullet ]} = \dfrac{1}{\ b_\bullet }\, g_\bullet \big (\frac{\delta }{c_\bullet b_\bullet }\big ) \end{aligned}$$

at $E^*_{[\bullet ]}$, which is a continuous function of $b_\bullet$ on the above-mentioned finite range. Then from the above arguments, it holds that $P^*_{[\bullet ]}\rightarrow P^*_{[1\oplus \bullet ]}$ as $b_\bullet \rightarrow b_\bullet ^-+0$ or $b_\bullet \rightarrow b_\bullet ^+-0$. On the other hand, from Theorem 7, $P^*_{[\bullet ]}$ for $b_\bullet \in (b_\bullet ^-, b_\bullet ^+)$ is greater than $P^*_{[1\oplus \bullet ]}$. Therefore, $P^*_{[\bullet ]}$ must take the maximum for a specific value of $b_\bullet \in (b_\bullet ^-, b_\bullet ^+)$.
